# Supplementary material for: Notch2 controls developmental fate choices between germinal center and marginal zone B cells upon immunization
Source: Nat Commun. 2024 Mar 4;15:1960. doi: 10.1038/s41467-024-46024-1 (PMC10912316; doi:10.1038/s41467-024-46024-1)
Supplement: Supplementary file 1 — Supplementary Information [file 41467_2024_46024_MOESM1_ESM.pdf]

Supplementary information

Notch2 controls developmental fate choices between germinal center and marginal zone B cells upon immunization

Tea Babushku, Markus Lechner, Stefanie Ehrenberg, Ursula Rambold, Marc Schmidt-Supprian, Andrew J. Yates, Sanket Rane*, Ursula Zimber-Strobl*, Lothar J. Strobl*

*These authors equally supervised the work

Corresponding author: Ursula Zimber-Strobl

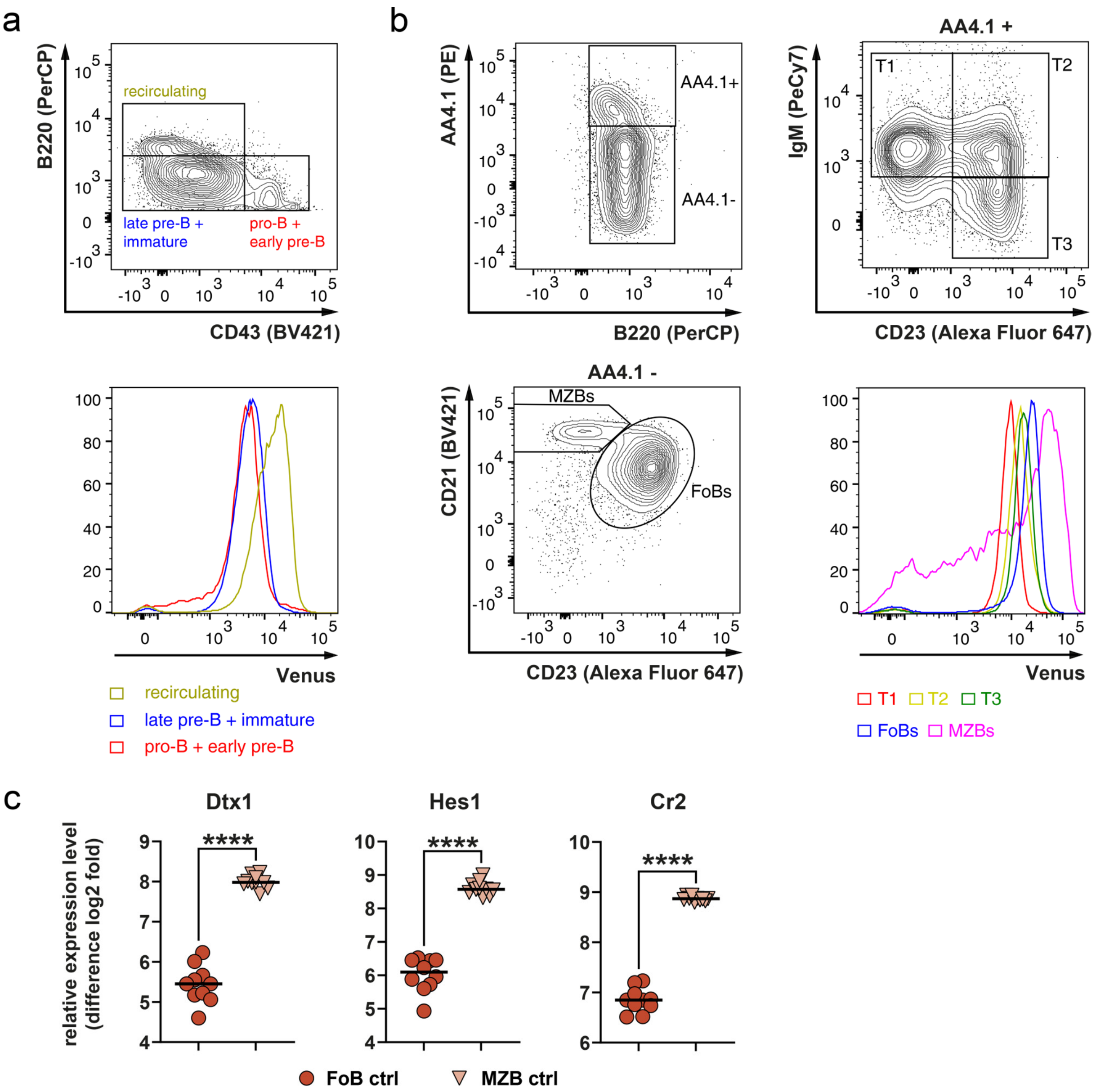

Supplementary Fig.1., related to Fig. 1. The H2B-Venus expression in B cells from CBF:H2B-Venus mice increases during B cell development (**a**) Representative FACS plot of B220^{+} lymphocytes from the bone marrow (BM) of CBF:H2B-Venus mice. B220^{+} cells were subdivided into pro- and early pre- (CD43^{+}B220^{low}) B cells (red), late pre- and immature B cells (CD43^{low}B220^{low}) (blue) and recirculating B cells (CD43^{-}B220^{high}) (yellow), n=5 mice. **(b)** Splenic transitional B cells were gated as AA4.1^{+}B220^{+} and subdivided into T1-T3 cells according to their differing CD23/IgM expression. Mature B cells were gated as AA4.1^{-}B220^{+} and further separated into Follicular B (FoB) (CD23^{+}CD21^{+}) and Marginal Zone B (MZB) cells (CD23^{low}CD21^{high}), n=5 mice. **(a+b)** The histogram overlays show the H2B-Venus expression in the previously gated cell populations (T1: red; T2: yellow; T3: green; FoB: blue; MZB: pink). (**c**) The graphs compile the relative expression levels of the known Notch target genes *Deltex1* (*Dtx1*), *Hes1*, and *Complement receptor 2 (Cr2 = CD21)* in FoB (dark orange dots) and MZB cells (light orange triangles) from control (CD19-Cre) mice, n=10. The scatter plots show individual data points with means. Paired two-tailed t-tests were performed (****$p < 0.0001$). The data are derived from the ENA dataset with the accession code PRJEB35207. Raw data from (c) are provided as a Source Data file.

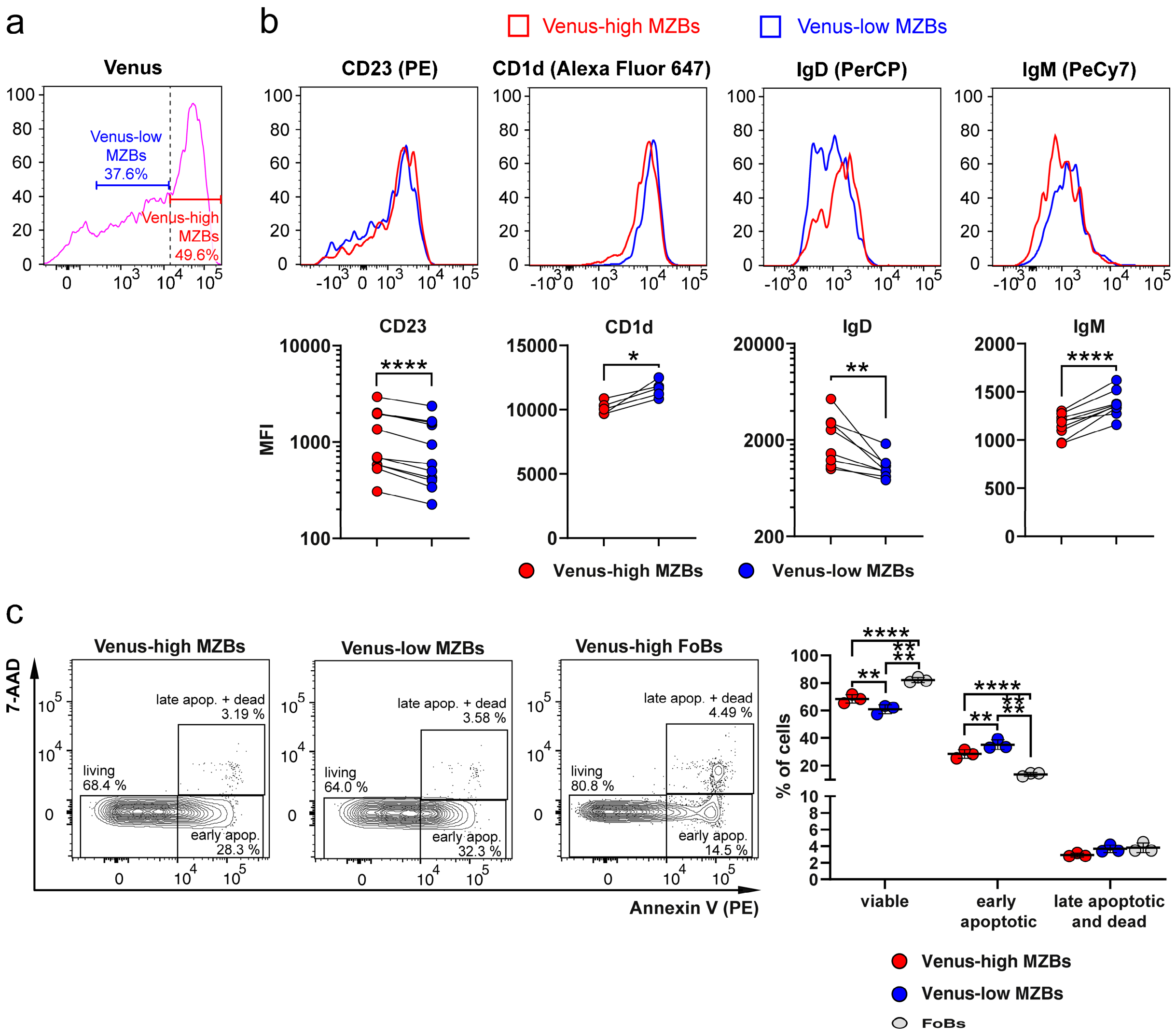

Supplementary Fig. 2, related to Fig. 2. H2B-Venus expression in Marginal Zone B cells from CBF:H2B-Venus mice is progressively downregulated during their further maturation. To further characterize H2B-Venus^{high} and H2B-Venus^{low} Marginal Zone B (MZB) cells, we assessed the expression of characteristic MZB cell markers as well as their apoptosis rate (Annexin staining). These findings (depicted in b-c) suggest that H2B-Venus^{low} cells have a more mature MZB cell phenotype in comparison to H2B-Venus^{high}cells. (**a**) The histogram shows the gating for H2B-Venus^{high} (red) and H2B-Venus^{low} MZB cells (blue). (**b**) H2B-Venus^{low} MZB cells have decreased levels of CD23 and IgD but increased levels of CD1d and IgM, compared to H2B-Venus^{high} cells. The histograms are pre-gated on B220^{+}CD21^{high}CD23^{low} MZB cells as shown in Fig. 1d. Representative histogram overlays of the expression of the indicated markers in H2B-Venus^{high} (red) and H2B-Venus^{low} (blue) MZB cells are shown. The graphs summarize the Median Fluorescence Intensity (MFI) of the indicated markers in the indicated populations from CD23: n=11 mice (7 males (m), 4 females (f)), CD1d: n=5 mice (3m, 2f), IgD: n=8 mice (4m, 4f) and IgM: n=9 mice (4m, 5f). After log transformation of data, paired two-tailed t-tests were performed (**** p<0.0001, *p=0.0111, **p=0.0072). **(c)** H2B-Venus^{low} cells display slightly elevated apoptosis rates compared to H2B-Venus^{high} cells. The graph illustrates the percentages of viable (AnnexinV^{-}7AAD^{-}), early apoptotic (AnnexinV^{+}7AAD^{-}) and late apoptotic/dead (Annexin^{+}7AAD^{+}) cells (gating is shown at the left side) in Venus-high (red) and Venus-low MZB cells (blue) and FoB cells (grey) from n=3 mice (3m). An ordinary two-way ANOVA with Tukey´s multiple comparison test was performed (**** p<0.0001; viable **p=0.0022; early apoptotic **p=0.0055). Raw data from (b) and (c) are provided as a Source Data file.

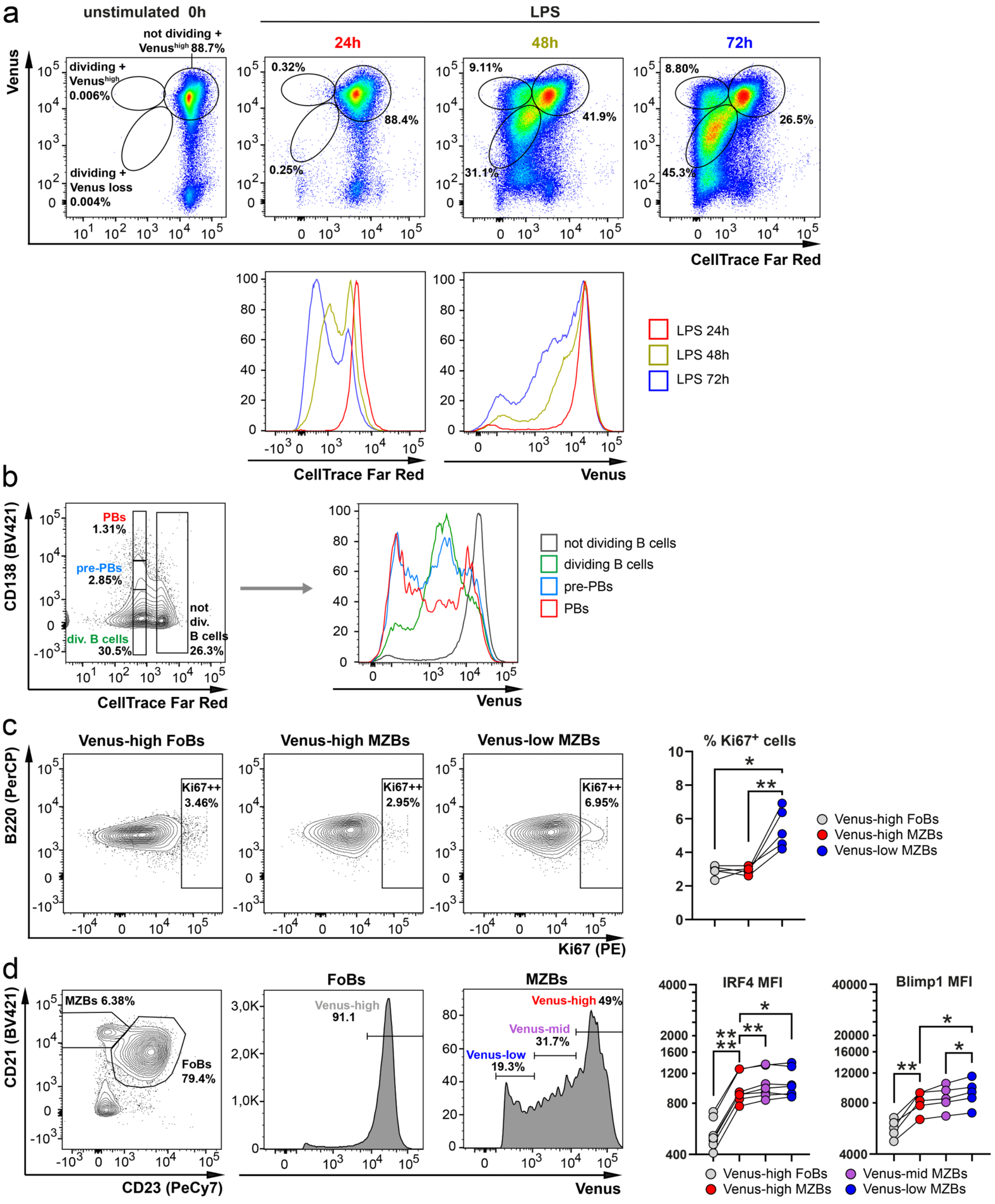

Supplementary Fig. 3, related to Fig. 2. H2B-Venus expression is lost during proliferation and plasma cell differentiation. a) Splenic B cells were stimulated with LPS for the indicated time points. To examine the correlation between H2B-Venus expression and cell division, we plotted the proliferation dye CellTrace Far Red against H2B-Venus. The gating strategy involved categorizing the cells into non-proliferating H2B-Venus^{high} cells (non-division + Venus-high), cells that remained H2B-Venus^{high} despite proliferation (division + Venus-high) and cells that divided while losing their H2B-Venus expression (division + Venus loss). The plots are gated on single living cells. In the lower row, histogram overlays for CellTrace Far Red and H2B-Venus at different time points of LPS

stimulation are presented (red 24h, yellow 48h, blue 72h). **b)** The FACS plot on the left illustrates the gating strategy, where cells (72h after LPS treatment) are initially gated based on equal CellTrace Far Red intensity and subsequently subdivided into $CD138^{low}$ cells (div. B cells), $CD138^{mid}$ cells (pre-plasmablasts (pre-PB)), and $CD138^{high}$ cells (PB). At the right side a histogram overlay shows the H2B-Venus expression in the respective subpopulations (non-dividing cells: black; dividing cells: green; pre-PBs: blue; PBs: red). **(c)** Ki-67^{+} cells were determined by using flow cytometry and a gating strategy as shown in the FACS plots. The graph summarizes the percentages of Ki-67^{+} cells in the indicated populations (Venus-high FoBs: grey; Venus-high MZBs: red; Venus-low MZBs: blue). n=5 mice (1 male, 4 female). Data were logtransformed before analysis. RM one-way ANOVA, Tukey´s multiple comparison test (*p=0.0227; **p=0.0076). **d)** The FACS histograms depict the H2B-Venus expression in Follicular B (FoB) cells ($CD23^{+}CD21^{high}$) and Marginal Zone B (MZB) cells ($CD23^{low}CD21^{high}$ cells). The gating strategy for FoB and MZB cells is presented in the contour plot on the left side. The gating of H2B-Venus^{high} FoB cells is indicated in the left histogram plot and of H2B-Venus^{high}, H2B-Venus^{mid} and H2B-Venus^{low} MZB cells in the right histogram. The graphs on the right side compile the Median Fluorescence Intensity (MFI) of Irf4 and Blimp1 in the indicated populations (Venus-high FoBs: grey; Venus-high MZBs: red; Venus-mid MZBs: purple; Venus-low MZBs: blue). After logtransformation of data RM one-way ANOVAs, Tukey´s multiple comparisons tests were applied (Irf4: n=7 mice (3 males, 4 females), ****p<0.0001, **p=0.003, *p=0.0249; Blimp1: n=5 mice, **p=0.005, left *p=0.0363, right *p=0.015). Raw data from (c) and (d) are provided as a Source Data file.

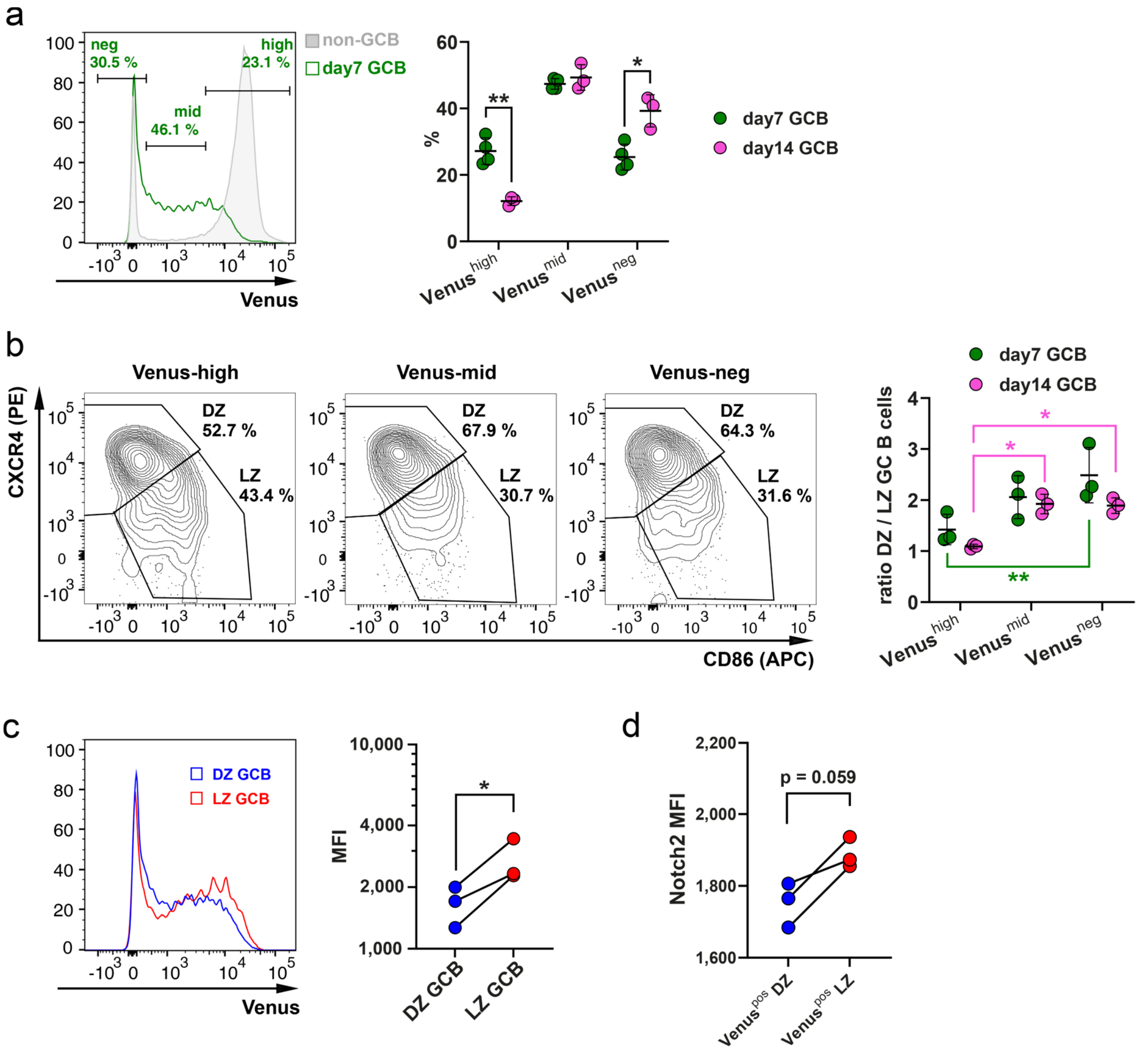

Supplementary Fig. 4, related to Figure 3. H2B-Venus expression in the light zone and dark zone of splenic Germinal Center B cells. (a) The histogram shows the overlay of H2B-Venus expression in Germinal Center B (GCB) (green) and non-GCB cells (filled grey) from CBF:H2B-Venus mice at day 7 post-immunization (p.i.) and the gating of H2B-Venus^{neg}, H2B-Venus^{mid} and H2B-Venus^{high} GCB cells. The graph depicts the percentages of the 3 subpopulations at indicated time points p.i.. RM two-way ANOVA, Sidak's multiple comparisons test (**p=0.0078 and *p=0.0498). d7 (green): n=4 mice (1 male, 3 females); d14 (purple): n=3 mice (1 male, 2 females). **(b)** The H2B-Venus^{high}, H2B-Venus^{mid}, and H2B-Venus^{low} GCB cells from (a) were analyzed for their dark zone (DZ) (CXCR4^{high}CD86^{low}) and light zone (LZ) (CXCR4^{low}CD86^{high}) distribution. FACS plots are representative for d7 p.i.. The graph depicts the ratio of the percentages of DZ-GCB to LZ-GCB cells for the three H2B-Venus subpopulations at d7 (green) and d14 (purple) p.i.. A lower DZ to LZ ratio suggests an enrichment of the indicated H2B-Venus subpopulation in the LZ. Ordinary two-way ANOVA, Sidak's multiple comparisons test (**p=0.0049; *p=0.0243 (left) and *p=0.0302 (right)), d7 and d14: n=3 mice (1 male, 2 females). (**c**) Representative overlay of the H2B-Venus expression in DZ-GCB and LZ-GCB cells. The graph summarizes the Median Fluorescence Intensity (MFI) of H2B-Venus in DZ-GCB (blue) and LZ-GCB (red) cells. After log transformation, a paired two-tailed t-test was performed (*p=0.03). n=3 mice (3 females) (**d**) The graph summarizes the Notch2 MFIs in DZ-GCB (blue) and LZ-GCB (red) cells. A paired two-tailed t-test was performed (p=0.059), n=3 mice (3 females). Raw data from (a-d) are provided as a Source Data file.

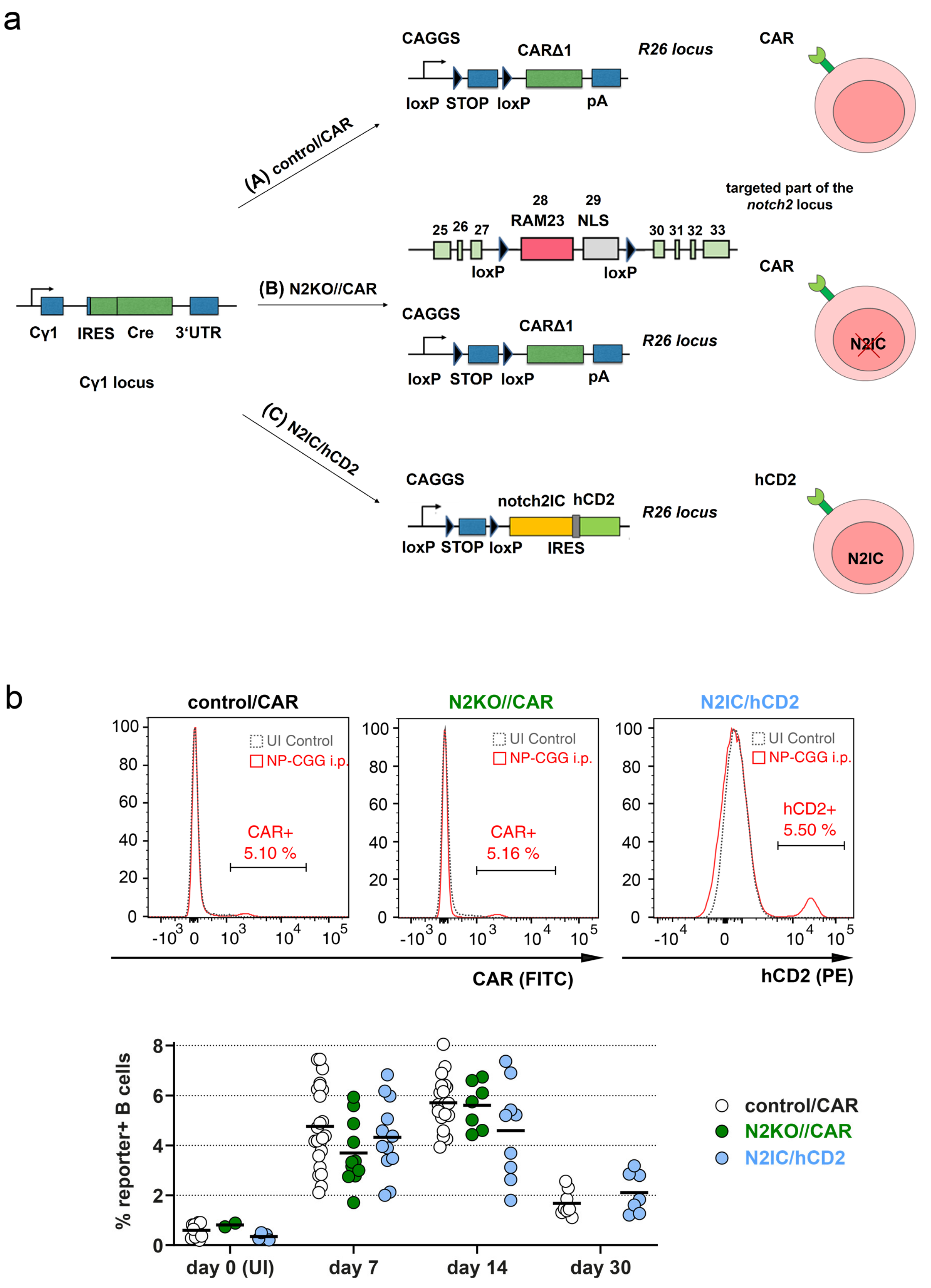

Supplementary Fig. 5, related to Figure 3. Mouse models for antigen-dependent inactivation or constitutive activation of Notch2. (a) Conditional Notch2$^{fl/fl}$ (1) or Notch2IC^{stopfl} (2) mice were crossed with the Cγ1-Cre mice (3). As controls, we used R26-CARΔ1^{stopF} mice (4). (A) Control/CAR: Cre recombinase activity leads to deletion of the loxP-flanked stop-cassette upstream of the *CarΔ1* transgene, resulting in expression of a truncated version of Cre-reporter coxsackie/adenovirus receptor (CAR) on the cell surface. (B) N2KO//CAR: Notch2$^{fl/fl}$ mice were combined with R26-CARΔ1^{stopF} (control/CAR) mice. In the targeted part of the *Notch2* locus, exons 28 and 29 coding for

the intracellular RAM23 domain and nuclear localization signal, respectively, are flanked with loxP sites. Upon Cre-mediated recombination, both the floxed *Notch2* exons and the stop-cassette upstream of *CarΔ1* in the *Rosa26* locus are excised, resulting in the inactivation of Notch2 and expression of the Cre-reporter CAR on the cell surface. (C) N2IC/hCD2: the coding sequence for *Notch2IC* is inserted downstream of a loxP flanked stop-cassette into the *Rosa26* locus. An internal ribosomal entry site (IRES element) and the transgene *hCD2* coding for a truncated version of the human CD2 receptor are inserted downstream of the *Notch2IC*. Cre-mediated excision results in expression of Notch2IC inside the cell and the Cre-reporter hCD2 on the cell surface. **(b)** Cre-reporter gene expression after immunization with NP-CGG: Histograms are pre-gated on living $B220^+$ lymphocytes. The histogram overlay illustrates the reporter expression in splenic B cells from unimmunized (UI) (dotted blue line) and immunized mice (red line) with the indicated genotypes**.** Gating of $reporter^+$ B cells is indicated in the histogram overlays and percentages of Cre-$reporter^+B220^+$ cells at indicated time points p.i. are summarized in the graph. Control/CAR mice (white dots): d0 n=11 (7 males (m), 4 females (f)), d7 n=23 (13m, 10f), d14 n=20 (11m, 9f), d30 n=8 (4m, 4f); N2KO//CAR mice (green dots): d0 n=2 (2m), d7 n=11 (3m, 8f), d14 n=7 (6m, 1f); N2IC/hCD2 mice (blue dots): d0 n=5 (3m, 2f), d7 n=12 (8m, 4f), d14 n=9 (5m, 4f), d30 n=7 (6m, 1f). Statistical analyses were performed for each time point separately. For day 0, day 7 and day 14 ordinary one-way ANOVAs with Tukey´s multiple comparison test and for day 30 an unpaired two-tailed t-test were performed. No significant differences were detected within the same time points. Raw data from (b) are provided as a Source Data file.

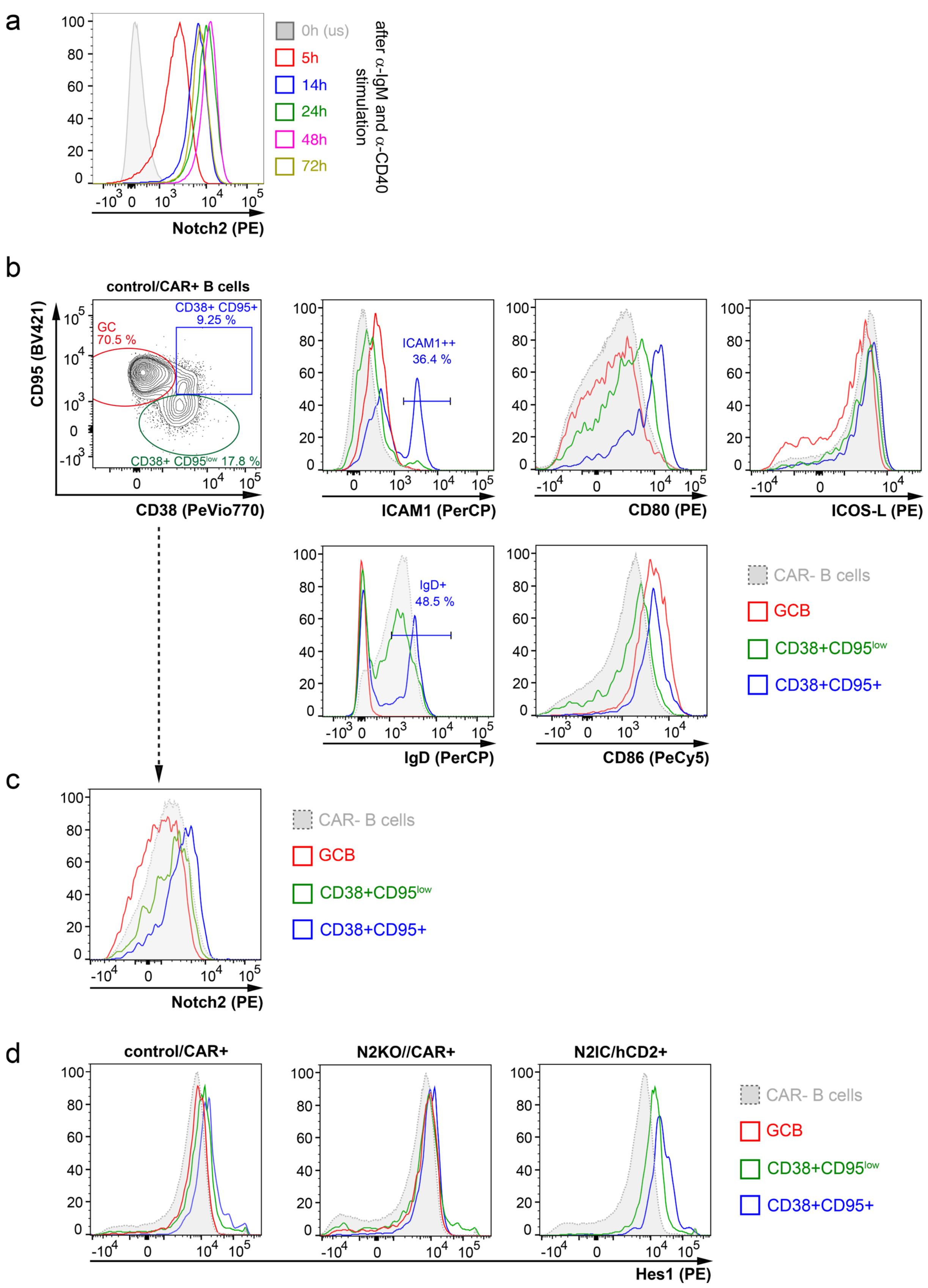

Supplementary Fig. 6, related to Figure 4. Notch2 surface expression and signaling on activated B cells. (a) An exemplary histogram overlay is shown, depicting the Notch2 expression on the cell surface of isolated Follicular B (FoB) cells at indicated time points after in vitro stimulation

with α-CD40+α-IgM. 0 h, unstimulated (us) FoB cells. Analysis is representative for n=5 mice, except for t=5h n=2 mice. The color coding of the different time points is indicated at the right side of the histogram. **(b)** The FACS plot shows the gating strategy for CAR^+ GCB cells ($CD95^{high}CD38^{low}$, red gate), activated B cells ($CD95^{high}CD38^+$, blue gate) and non-GCB cells ($CD95^{low}CD38^+$, green gate) in splenic B cells from control/CAR mice 14d post-immunization (p.i.). The plot is pre-gated on CAR^+B220^+ lymphocytes. The histograms show overlays of the indicated surface markers in the indicated populations (CAR^- B cells: filled grey; GCB cells: red; non-GCB cells: green; activated B cells: blue). ICAM-1, CD80 and CD86 are upregulated in $CD38^+CD95^+$ cells in comparison to CAR^- cells suggesting that these cells are activated. The bimodal expression of ICAM-1 and IgD suggests that the $CD38^+CD95^+$ fraction contains both activated FoB and memory B cells. **(c)** The histogram overlay shows the Notch2 cell surface expression in the indicated cell subpopulations (CAR^- B cells: filled grey; GCB cells: red; non-GCB cells: green; activated B cells: blue) in the indicated genotypes at day 14 p.i.. Representative for n=6 mice. **(d)** Histogram overlays of the Hes1 expression in the indicated cell populations (CAR^- B cells: grey; GCB cells: red; non-GCB cells: green; activated B cells: blue) and genotypes. GCB cells were excluded in the histogram overlays from N2IC/hCD2 mice due to lack of GCB cells in this genotype. Representative for n=4 mice.

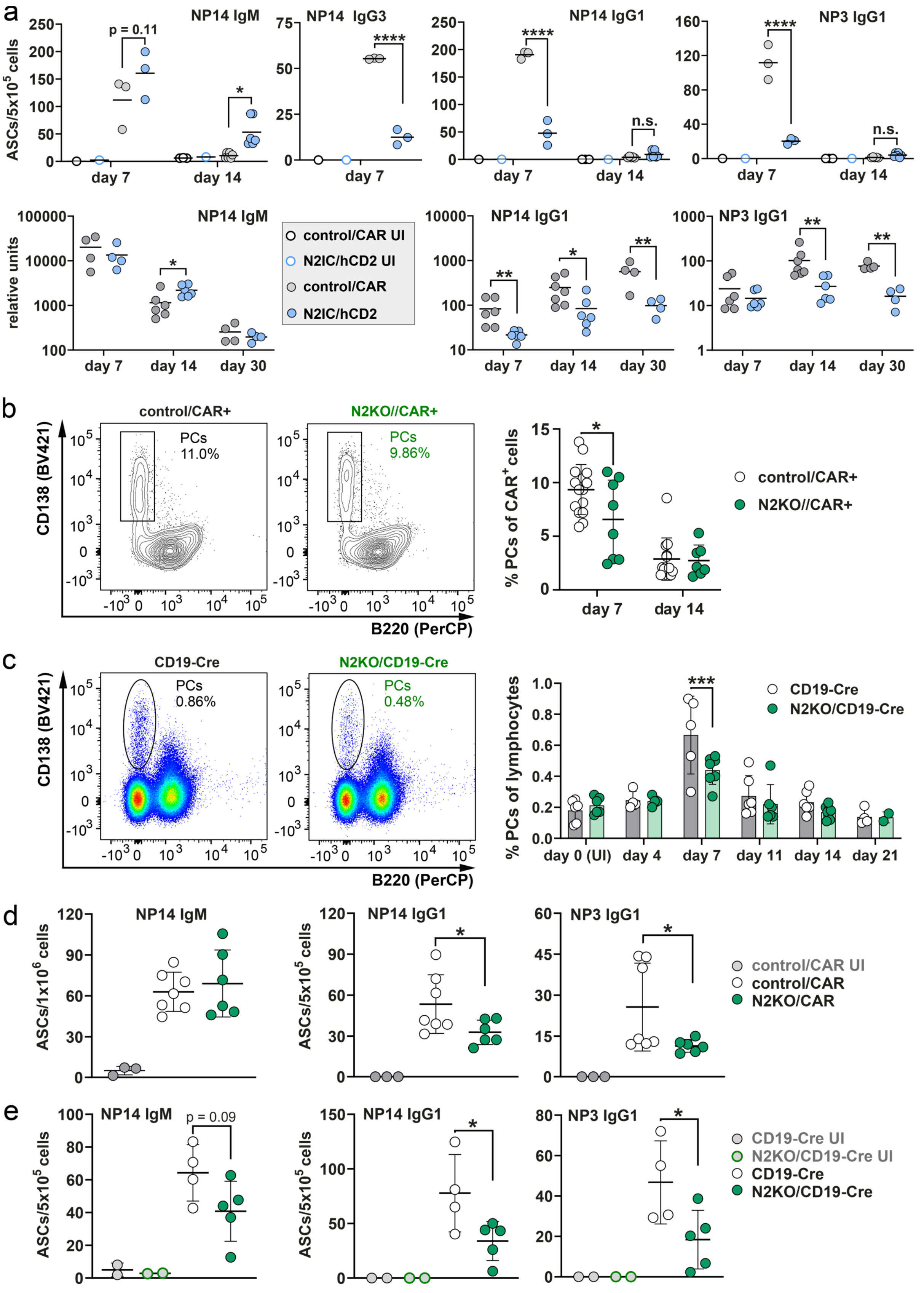

Supplementary Fig. 7, related to Figure 5. Notch2 signaling must be downregulated to enable terminal plasma cell differentiation. (a) Total (NP14-BSA) and high affinity (NP3-BSA) antibody-

secreting cells (ASC) of the indicated isotypes were determined in the spleen of mice at d7 and d14 post-immunization (p.i.) (upper row). Control/CAR mice unimmunized (UI) (white dots): d7 n=1 (1female (f)), d14 n=3 (1 male (m), 2 f); immunized control/CAR mice (grey dots): d7 n=3 (3f), d14 n=6 (4m, 2f); N2IC/hCD2 mice UI (white dots with blue outline): d7 n=1 (1m), d14 n=1 (1f); immunized N2IC/hCD2 mice (blue dots): d7 n=3 (3m), d14 n=6 (4m, 2f). Except for IgG3 (unpaired two-tailed t-test), ELISpot data were analyzed by ordinary two-way ANOVAs with Sidak´s multiple comparison test (*p=0.0453 and ****p<0.0001). In addition, NP-specific serum titers of IgM and IgG1 were determined (lower row). IgM: Control/CAR mice (grey dots): d7 n=4 (3m, 1f), d14 n=6 (4m, 2f); d30 n=4 (1m, 3f); N2IC/hCD2 mice (blue dots): d7 n=4 (3m, 1f), d14 n=6 (2m, 4f), d30 n=4 (3m, 1f). IgG1: Control/CAR mice (grey dots): d7 n=6 (5m, 1f), d14 n=7 (4m, 3f); d30 n=4 (1m, 3f); N2IC/hCD2 mice (blue dots): d7 n=6 (5m, 1f), d14 n=6 (2m, 4f), d30 n=4 (3m, 1f). After log transformation, ELISA data were only compared within the respective days by unpaired two-tailed t-tests (IgM: *p=0.0177; NP14-IgG1: **p=0.003, *p=0.0168, **p=0.0096; NP3-IgG1: **p=0.0031, **p=0.0011). **(b)** The percentages of Cre-reporter$^+$ plasma cells (PC) (CD138$^+$B220$^-$) were determined at the indicated time points after immunization with NP-CGG. The FACS plots are representative for d7: n=15 (8m, 7f) and n=8 (3m, 5f); d14: n=13 (7m, 6f) and n=7 (6m, 1f) control/CAR mice (white dots) and N2KO//CAR mice (green dots), respectively; Data were statistically analyzed by an ordinary two-way ANOVA with Sidak´s multiple comparison test (*p=0.0245). **(c)** PCs (CD138$^+$B220$^-$) in N2KO//CD19-Cre and CD19-Cre mice p.i.. The FACS plots illustrates the gating, the graph compiles the percentages of PCs in CD19-Cre mice (grey bars) n=6 (2m, 4f), n=4 (2m, 2f), n=5 (1m, 4f), n=6 (4m, 2f), n=6 (4m, 2f), n=4 (3m, 1f) and N2KO//CD19-Cre mice (green bars) n=6 (4m, 2f), n=4 (2m, 2f), n=7 (3m, 4f), n=6 (2m, 4f), n=8 (5m, 3f), n=2 (1m, 1f) at days 0, 4, 7, 11, 14, and 21 p.i., respectively. Data were analyzed by an ordinary two-way ANOVA with uncorrected Fisher´s LSD (***p=0.0005). **(d-e)** Numbers of ASCs in the spleen from N2KO//CAR and control/CAR mice **(d)** and N2KO//CD19-Cre mice and CD19-Cre mice **(e)** at d7 p.i.. **(d)** unimmunized control/CAR (grey dots) n=3 (1m, 2f); immunized control/CAR mice (white dots) n=7 (2m, 5f); immunized N2KO//CAR mice (green dots) n=6 (4m, 2f). **(e)** unimmunized control/CAR (grey dots) n=2 (1m, 1f); unimmunized N2KO//CAR mice (grey dots with green outline) n=2 (2m), immunized control/CAR mice n=4 (4f) (white dots); immunized N2KO//CAR mice (green dots) n=5 (5m). **(d+e)** only data between immunized genotypes were compared by unpaired two-tailed t-tests (N2KO//CAR: *p=0.0356, *p=0.0394; N2KO//CD19-Cre: *p=0.0452, *p=0.0451). Raw data are provided as a Source Data file.

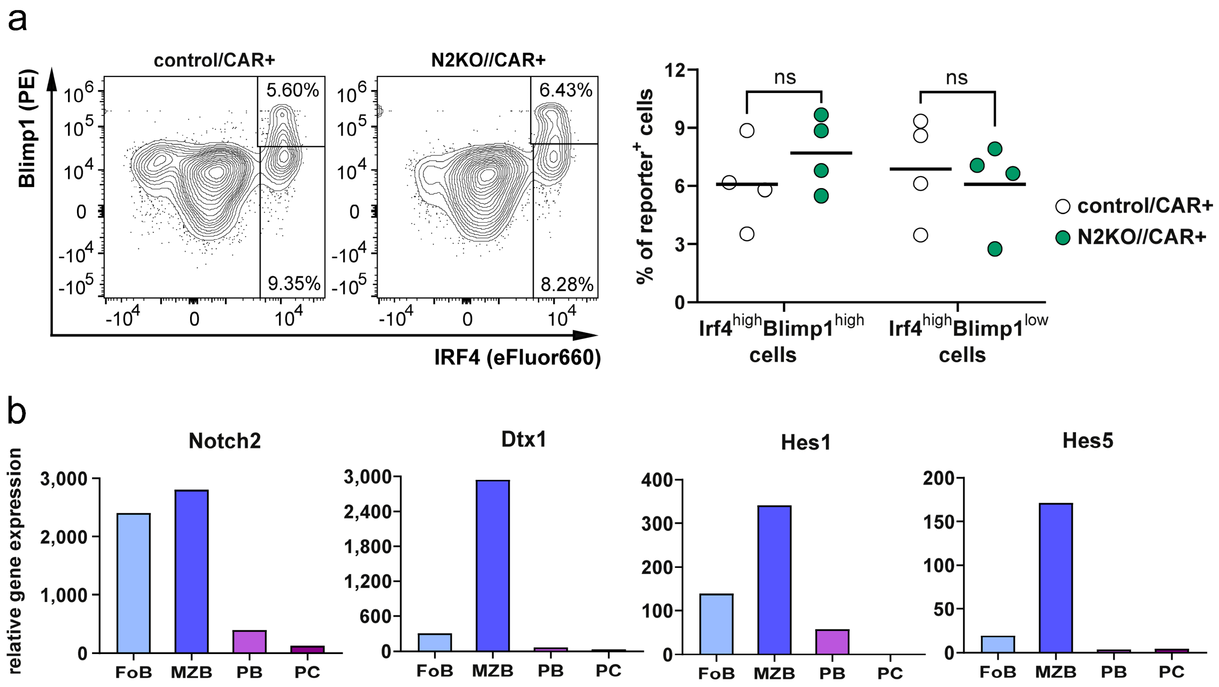

Supplementary Fig. 8, related to Figure 5. (a) Representative Irf4/Blimp1 FACS plots from d7 post-immunization (p.i.). Splenocytes were pre-gated on living Cre-reporter^{+} lymphocytes. The Irf4^{high} fraction was divided into a Blimp1^{low} fraction (pre-PB and early PB) and a Blimp1^{high} fraction (late plasmablasts (PB) and plasma cells (PC)). Percentages of Blimp1^{high} and Blimp1^{low} cells in the Irf4^{high} fraction from control/CAR (white dots) and N2KO//CAR (green dots) mice are summarized in the graph, n=4 mice (2m, 2f) for both genotypes. Data were analyzed by an ordinary two-way ANOVA with Sidak´s multiple comparison test. **(b)** In silico analysis of the mRNA expression of *Notch2* and the Notch target genes *Deltex1* (*Dtx1)*, *Hes1* and *Hes5* in the indicated populations (Follicular B cells (FoB): light blue; Marginal Zone B cells (MZB): dark blue; Plasmablasts (PB): light purple; Plasma cells (PC): dark purple) using the RNAseq Gene Skyline app from the ImmGen.org database (http://rstats.immgen.org/Skyline/skyline.html).

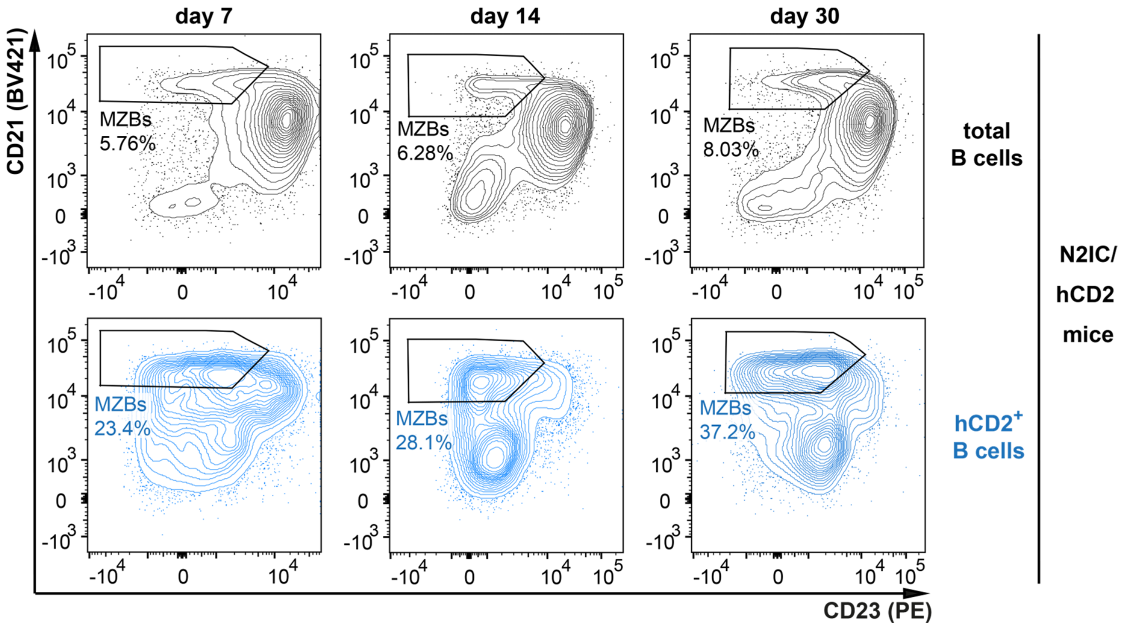

Supplementary Fig. 9, related to Fig. 6. CD23/CD21 staining to separate and quantify Marginal Zone B cells in N2IC/hCD2 mice. Representative FACS plots of the separation of $CD21^{high}CD23^{low}$ Marginal Zone B (MZB) cells among total $B220^{+}$ B cells (upper row, black) and $Cre\text{-}reporter^{+}$ B cells (lower row, blue) in N2IC/hCD2 mice at the indicated time points post immunization (p.i.). The analyses are representative for n=12 mice at day 7, n=9 mice at day 14 and n=7 mice at day 30.

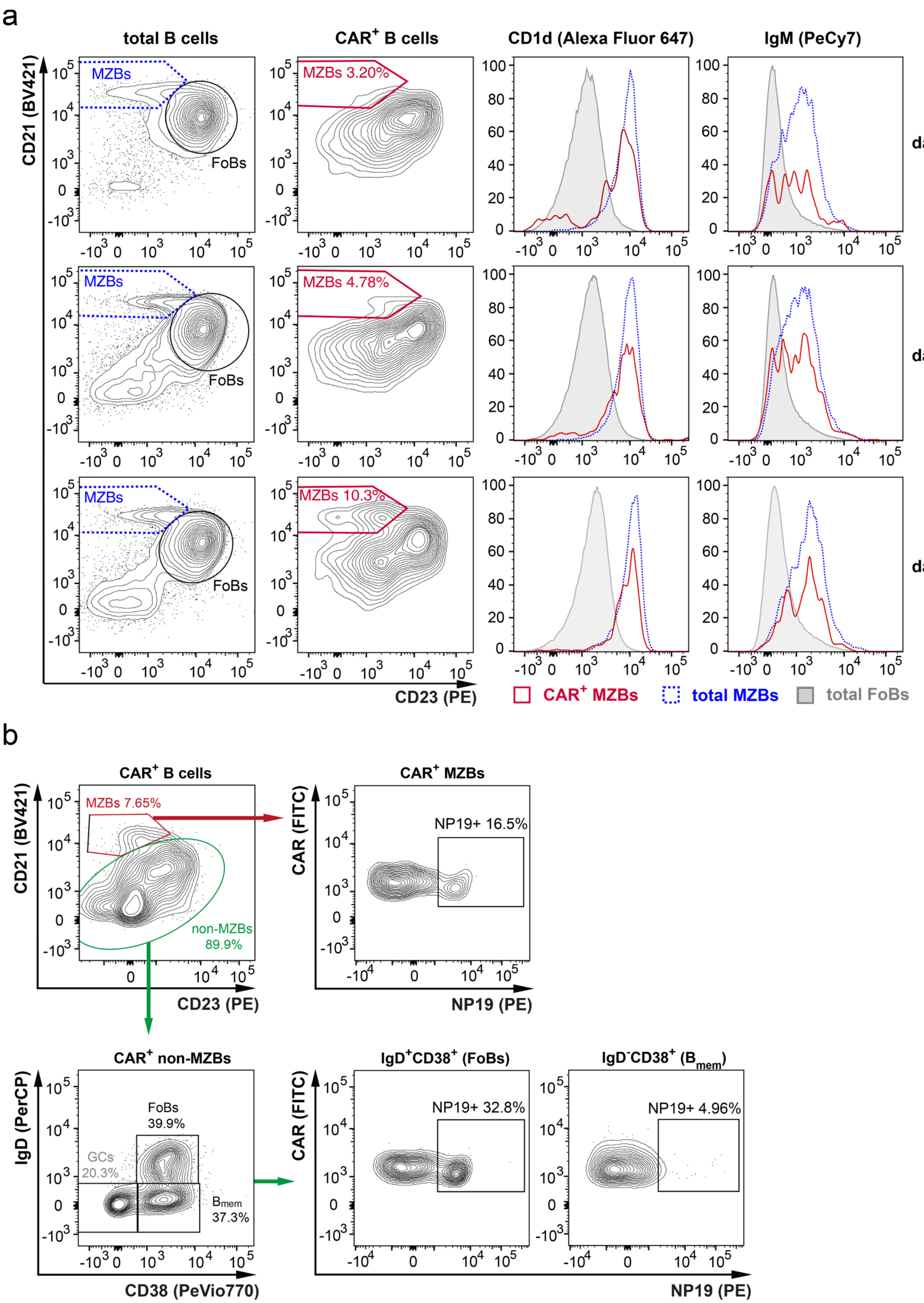

Supplementary Fig. 10, related to Figure 7. The differentiation of Marginal Zone B cells occurs physiologically during TD-immune responses. (a) Exemplary FACS plots to illustrate the gating of total Marginal Zone B (MZB) cells (CD23^{low}CD21^{high}) (blue gate, dotted line), total Follicular B

(FoB) cells ($CD23^{+}CD21^{mid/low}$) (black gate, solid line) and newly generated MZB cells ($CAR^{+}CD23^{low}CD21^{high}$) (red gate, solid line). The histogram overlays show cell surface expression levels of CD1d and IgM on the gated CAR^{+} MZB cell population (red, solid), compared to the total MZB (blue, dotted) and total FoB cells (grey, filled) in control/CAR mice at indicated time points post TD-immunization with NP-CGG (p.i.). The analyses are representative for n=16 mice at d7, n=17 mice at d14 and n=8 mice at d30. **(b)** Gating strategy for the determination of NP^{+} cells within the indicated subpopulations. CAR^{+} cells were divided into MZB cells ($CD21^{high}CD23^{low}$) (red gate) and non-MZB cells ($CD21^{+}CD23^{+/low}$) (green gate). Non-MZB cells were further subdivided into $CD38^{-}$ IgD^{-} GCB cells, $CD38^{+}IgD^{+}$ FoB cells and $CD38^{+}IgD^{-}$ memory B cells. The percentage of NP-specific cells was determined in each population and afterwards calculated back to the total percentage of CAR^{+} cells.

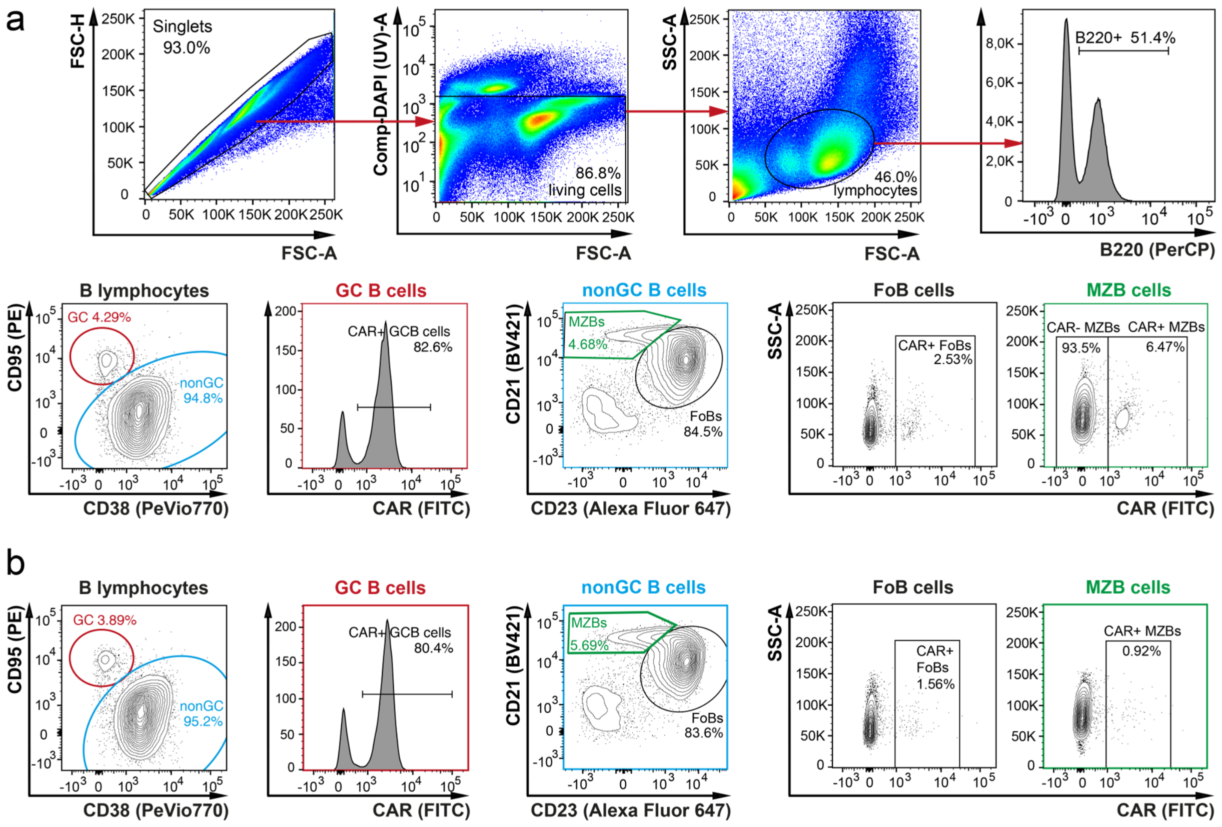

Supplementary Fig. 11, related to Figure 8-9. Sequential gating for CAR-expressing cells in different B cell subpopulations for mathematical modelling. **(a-b)** FACS plots are representative for d7 post-immunization with NP-CGG (p.i.). **(a)** Hierarchical gating of splenocytes in control/CAR mice to retrieve the percentages of total living B220^{+} B cells, CAR^{+} Germinal Center B (GCB) cells (CD38^{low}CD95^{high}) (within red gate) and non-GCB cells (CD38^{+}CD95^{low}) (within blue gate). The frequencies of CAR^{+} GCB cells were gated in histograms. The non-GC B cell fraction was further subdivided into Marginal Zone B (MZB) (CD23^{low}CD21^{high}) (green gate) and Follicular B (FoB) cells (CD23^{+}CD21^{low}) (black gate). Within MZB and FoB cells the percentages of CAR-expressing cells were gated in CAR vs. SSC-A plots. The frequencies of CAR^{-} cells within the non-GC MZB cell fraction and the total non-GC FoB cells were used for generating the plots in Supplementary Fig. 12. The gating strategy is representative for the following mouse numbers: n=3 at d4, n=9 at d7, n=3 at d9, n=15 at d14, n=3 at d17, n=5 at d22, n=4 at d26, n=6 at d30 p.i.. **(b)** Exemplary analysis of splenocytes from N2KO//CAR mice. Singlets, living cells, lymphocytes, Cre-reporter^{+} GCB cells and Cre-reporter^{+} non-GC FoB and MZB cells were sequentially gated as in **(a)**. The gating strategy is representative for the following mouse numbers n=7 at d7, n=5 at d9, n=6 at d14 p.i.. Raw data are provided as a Source Data file (see sheets modeling data).

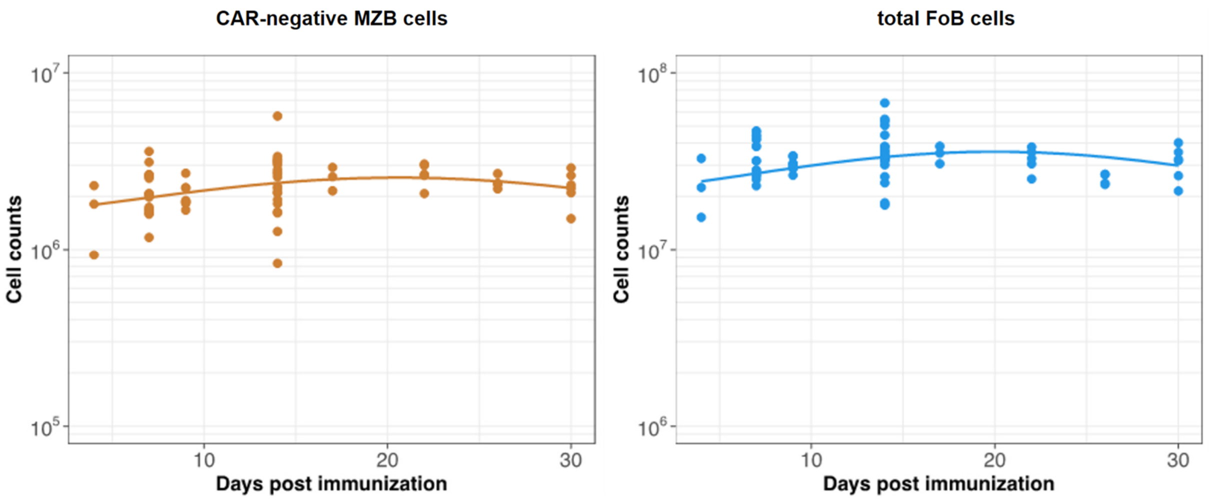

Supplementary Fig. 12, related to Fig. 9. Empirical descriptions of the dynamics of the numbers of Follicular B cells and Marginal Zone B cells. We show here curves for total Follicular B (FoB) cells and CAR^{-} Marginal Zone B (MZB) cells using the functions (defined in Supplementary Note 2), which we use as inputs in our ODE systems. The y-axis depicts the total cell numbers of the respective cell population at d4 (n=3; 2 males (m), 1 female (f)), d7 (n=9, 5m, 4f), d9 (n=3; 2m, 1f), d14 (n=15; 8m, 7f), d17 (n=3; 3f), d22 (n=5; 1m, 4f), d26 (n=4; 2m, 2f), d30 (n=6; 4m, 2f) in control/CAR mice, following immunization. Raw data are provided as a Source Data file (see sheets modeling data).

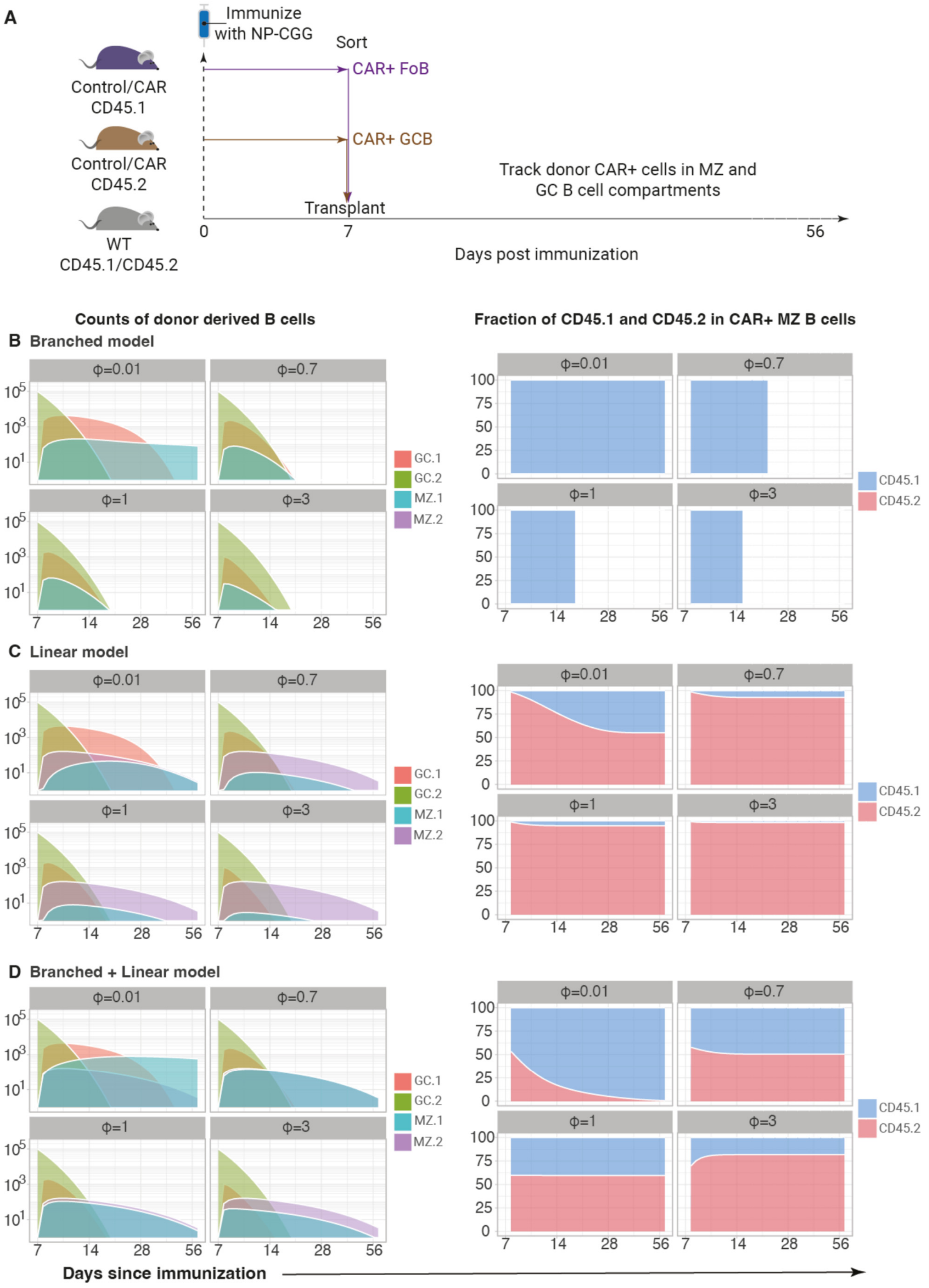

Supplementary Fig. 13. Simulation of the co-transfer experiment using parameters derived from modeling the B cell response to immunization in control/CAR mice. (A) Schematic of the proposed co-transfer experiment. **(B-D)** Results from the simulations of (B) branched, (C) linear and

(D) branched + linear models of Marginal Zone B (MZB) cell generation during an active immune response. Plots on the left show simulated cell counts of Germinal Center (GC.1) and Marginal Zone (MZ.1) B cells from CD45.1 mice and Germinal Center (GC.2), and Marginal Zone (MZ.2) B cells from CD45.2 donors. Plots on the right depict simulated fractions of CD45.1 and CD45.2 donor cells in CAR^+ MZB cell compartment. Each panel shows the predictions generated using a fixed value of the loss rate (ϕ) of CAR^+ Follicular B cells, as described in the Supplementary Note 3.

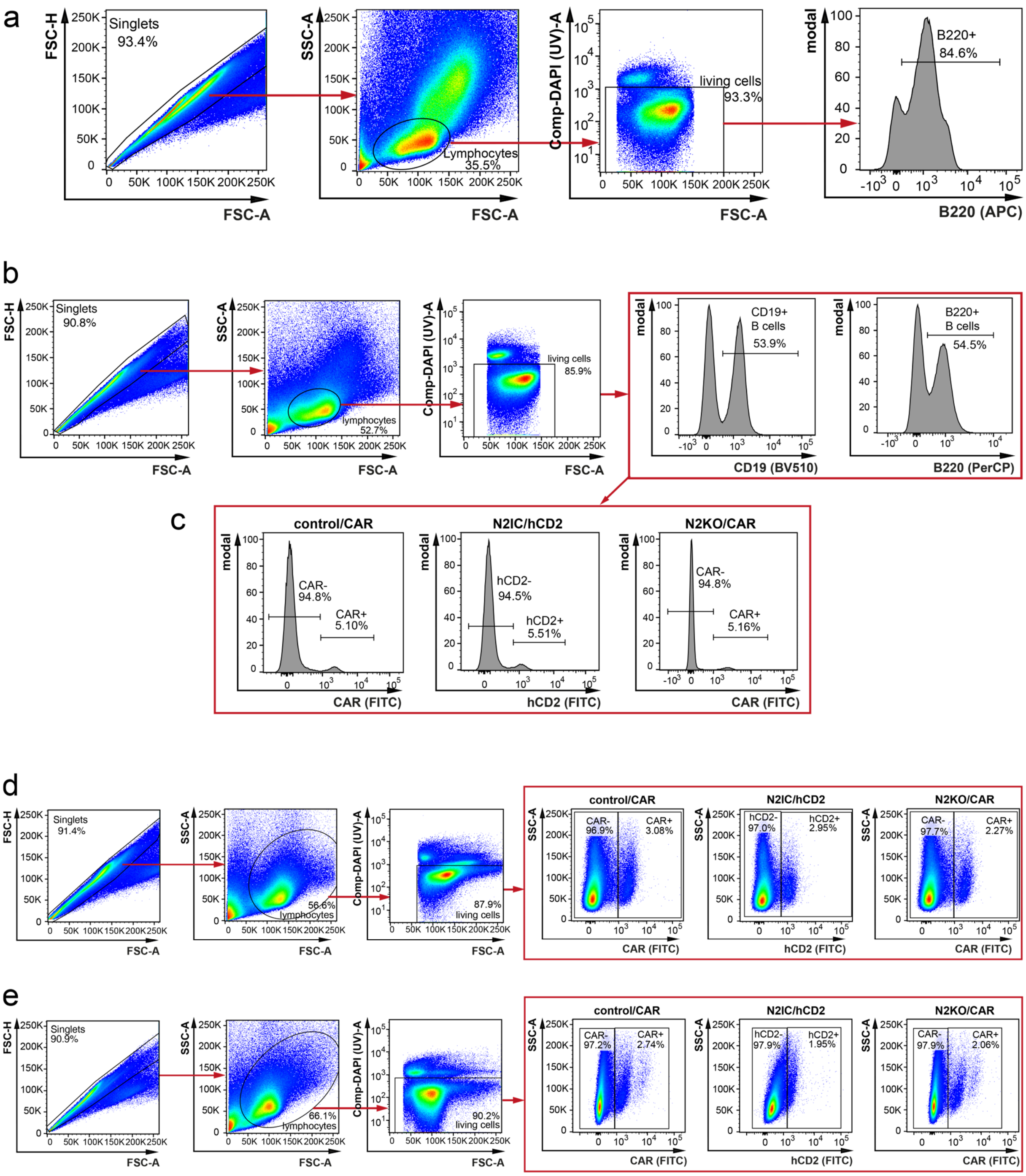

Supplementary Figure 14. Additional representative flow cytometry gating strategies for extracellular and intracellular cell stainings. (a) Sequential gating strategy for living B220^{+} B lymphocytes in the BM used for Supplementary Fig. 1a. **(b)** Sequential gating strategy for living B220^{+} or CD19^{+} B cells in the spleen **(c)** B220^{+} B cells were further subdivided in reporter^{+} and reporter^{-} cells. This sequential gating was used for the extracellular staining in Fig. 2c, 3a, 3c, 3f, 4d, 6e, 7a, Supplementary Fig. (S)1b, S2c, S5b, S6b, S9 and S10 and the intracellular staining in Fig. 1d, 2a, 4c, S3c, S3d and S6d. (**d-e**) Sequential gating strategy with a large lymphocyte gate to include plasmablasts and plasma cells among living total or reporter^{+} lymphocytes in the spleen. This gating strategy was used for the extracellular staining **(d)** and intracellular staining **(e)**, used for Fig. 4f, 4g, 5, 6a, S7b, S7c and S8a.

Supplementary Table 1. List of antibodies used in this study.

**Flow Cytometry**						
Antibody	Conjugate	Clone	Company	Catalog No.	Dilution	RRID / access link for validation data
Anti-Human CD2 (hCD2)	APC	RPA-2.10	eBioscience	17-0029-42	1:100	AB_10805740
Anti-Human CD2 (hCD2)	PE	RPA-2.10	eBioscience	12-0029-42	1:100	AB_10670621
Anti-Human CD2 (hCD2)	FITC	REA972	Miltenyi Biotec	130-116-251	1:50	AB_2727364
Anti-human CD2 (hCD2)	Pe Vio770	REA972	Miltenyi Biotec	130-116-254	1:100	AB_2727364
Anti-Human CAR	FITC	E1-1	Santa Cruz	sc-56892 FITC	1:25	10.3390/cells11050841; 10.1182/blood.2018880138;
Anti-Mouse CD1d	Alexa Fluor 647	1B1	BioLegend	123511	1:200	AB_1236539
Anti-Mouse TACI	APC	ebio8F10-3	eBioscience	17-5942	1:100	AB_842758
Anti-Mouse CD86	APC	GL1	BD Biosciences	561964	1:100	AB_2075114
Anti-Mouse CD184 (CXCR4)	PE	2B11	eBioscience	12-9991-82	1:400	AB_891391
Anti-Mouse CD38	Pe Vio770	REA616	Miltenyi Biotec	130-125-522	1:100 (1:50 for IC)	AB_2802049
Anti-Mouse CD21/CD35	BV421	7G6	BD Biosciences	562756	1:200	AB_2737772
Anti-Mouse CD97 (AA4.1)	PE	AA4.1	eBioscience	12-5892	1:200	AB_466018
Anti-Mouse CD138	BV421	281-2	BD Biosciences	562610	1:200	AB_11153126
Anti-Mouse CD95	BV421	Jo2	BD Biosciences	562633	1:200 (1:100 for IC)	AB_2737690
Anti-Mouse CD95	PE	Jo2	BD Biosciences	554258	1:300	AB_395330

Anti-Mouse Bcl-6	Alexa Fluor 647	K112-91	BD Biosciences	561525	1:50	AB_10898007
Anti-Mouse PRDM1/Blimp-1	PE	6D3	Santa Cruz	sc-47732 PE	1:50	https://datasheets.scbt.com/sc-47732.pdf
Anti-Mouse IRF4	eFluor 660	3E4	eBioscience	50-9858-82	1:100	AB_2574393
Anti-Mouse IRF4	PE	3E4	eBioscience	12-9858-82	1:500	AB_10852721
Anti-Mouse Notch2	PE	HMN2-35	BioLegend	130707	1:80	AB_1227725
Anti-Mouse CD43	Biotin	S7	BD Biosciences	553269	1:350	AB_2255226
Anti-Mouse CD43	BV421	S7	BD Biosciences	562958	1:200	AB_2665409
Anti-Mouse CD19	Pe Vio770	REA749	Miltenyi Biotec	130-112-037	1:200	AB_2655830
Anti-Mouse CD19	BV510	1D3	BD Biosciences	562956	1:200	AB_2737915
Anti-Mouse CD45R/B220	PerCP	RA3-6B2	BD Biosciences	553093	1:100	AB_394622
Anti-Mouse CD45R/B220	APC	RA3-6B2	BD Biosciences	553092	1:250	AB_398531
Anti-Mouse CD45R/B220	PE	RA3-6B2	BD Biosciences	553090	1:350	AB_394619
Anti-Mouse CD45R/B220	FITC	RA3-6B2	BD Biosciences	553088	1:200	AB_394618
Streptavidin	PerCP	N/A	BD Biosciences	554064	1:100	AB_2336918
Streptavidin	APC	N/A	BD Biosciences	554067	1:400	AB_10050396
Anti-Mouse CD23	PE	B3B4	BD Biosciences	553139	1:200	AB_394654
Anti-Mouse CD23	Alexa Fluor 647	B3B4	BD Biosciences	562826	1:100	AB_2737821
Anti-Mouse CD23	PE-Cy7	B3B4	BD Biosciences	562825	1:200	AB_2737820

Anti-Mouse IgM	APC	II/41	BD Biosciences	562032	1:100	AB_398464
Anti-Mouse IgM	PE-Cy7	R6-60.2	BD Biosciences	552867	1:300	AB_394500
Anti-Mouse IgD	Biotin	11-26c	eBioscience	13-5993-82	1:350	AB_466860
Anti-mouse IgG1	PE	A85-1	BD Biosciences	550083	1:800	AB_393553
Anti-mouse Ki-67	PE	SolA15	eBioscience	12-5698-82	1:500	AB_11150954
Anti-mouse CD54 (ICAM-1)	Biotin	3E2	BD Biosciences	553251	1:200	AB_394733
Anti-mouse CD275 (ICOS-L)	PE	HK5.3	eBioscience	12-5985-82	1:100	AB_466094
Anti-mouse CD80	PE	16-10A1	eBioscience	12-0801-82	1:400	AB_465752
Anti-mouse CD86	Pe-Cy5	GL1	eBioscience	15-0862-82	1:200	AB_468778
NP (4-Hydroxy-3-nitrophenylacetyl hapten) 19	PE	N/A	LGC Biosearch Technologies	N-5070-1-BS	1:200	https://shop.biosearchtech.com/immunochemicals/hapten-conjugates/np-pe-%28phycoerythrin%29/p/N-5070-1#specifications
Anti-Mouse CD184 (CXCR4)	BV510	2B11	BD Biosciences	563468	1:200	AB_2738225
Rabbit anti-Hes1 monoclonal antibody	unconjugated	D6P2U	Cell Signaling	11988S	1:100	https://www.cellsignal.com/datasheet.jsp?productId=11988&images=1&size=A4
Goat anti-Rabbit IgG (H+L) cross-adsorbed	PE	N/A	Invitrogen	P-2771MP	1:100	AB_2539845

Annexin V Apoptosis Detection Kit I	PE	N/A	BD Biosciences	559763	5 µl per 1 million cells in 100 µl	AB_2869265
CellTrace™ Far Red Cell Proliferation Kit	FarRed (APC)	N/A	Invitrogen	C34564	1:1000	https://www.thermofisher.com/order/catalog/product/de/en/C34564
**Enzyme-linked Immunosorbent Assay (ELISA)**						
rat-anti-mouse IgM	HRP	YF97	Southern Biotech	1140-05	1:5000	AB_2794629
rat-anti-mouse IgG1	Biotin	A85-1	BD Biosciences	553441	1:500	AB_394861
Avidin D	HRP	N/A	Vector	A-2014-5	1:2000	https://vectorlabs.com/products/avidin-d-horseradish-peroxidase-concentrate-elisa
**Enzyme-linked immunosorbent spot (ELISpot)**						
Anti-Mouse IgM	Biotin	R6-60.2	BD Biosciences	553406	1:500	AB_394843
rat-anti-mouse IgG1	Biotin	A85-1	BD Biosciences	553441	1:500	AB_394861
Anti-Mouse IgG3	Biotin	R40-82	BD Biosciences	553401	1:500	AB_394838
Avidin D	HRP	N/A	Vector	A-2014-5	1:2000	https://vectorlabs.com/products/avidin-d-horseradish-peroxidase-concentrate-elisa
**Histology (IF/IHC)**						
Rabbit anti-Laminin	unconjugated	L9393	Sigma-Aldrich	L9393	1:100	AB_477163
Rat anti-Human CD2 (hCD2)	Biotin	RPA-2.10	BD Biosciences	555325	1:50	AB_395732
Rat anti-Mouse CD90.2 (Thy1.2)	Biotin	30-H12	BD Biosciences	553011	1:100	AB_394549
Rat anti-Mouse CD45R/B220	APC	RA3-6B2	BD Biosciences	553092	1:500	AB_398531

Rat anti-Mouse T- and B-Cell Activation Antigen (GL7)	FITC	GL7	BD Biosciences	553666	1:100	AB_394981
Rat anti-Mouse T- and B-Cell Activation Antigen (GL7)	Alexa Fluor 647	GL7	BD Biosciences	561529	1:100	AB_10716056
Rat anti-Metallophilic Macrophages (MOMA-1)	Biotin	MOMA-1	Abcam	ab51814	1:100	https://www.abcam.com/products/primary-antibodies/biotin-metallophilic-macrophages-antibody-moma-1-ab51814.html
Rat anti-Mouse Irf4	unconjugated	3E4	eBioscience	14-9858-80	1:100	https://www.thermofisher.com/antibody/product/IRF4-Antibody-clone-3E4-Monoclonal/14-9858-82
Goat anti-Mouse IgM	unconjugated	1020-01	Southern Biotech	1020-01	1:100	AB_2794197
Chicken anti-Goat IgG	Alexa Fluor 647	N/A	Invitrogen	A-21469	1:500	AB_2535872
Goat anti-Rat IgG	Alexa Fluor 488	N/A	Jackson ImmunoResearch	112-545-003	1:500	AB_2338351
Goat anti-rabbit IgG	Cyanine Cy3	N/A	Jackson ImmunoResearch	111-165-003	1:500	AB_2338000
Goat anti-Rat IgG	Alexa Fluor 647	N/A	Jackson ImmunoResearch	112-605-167	1:500	AB_2338404
Anti-Rabbit IgG	HRP	N/A	Sigma-Aldrich	A0545	1:200	AB_1645286
Streptavidin	Alexa Fluor 594	N/A	Invitrogen	S11227	1:500	https://www.thermofisher.com/order/catalog/product/de/en/S11227

Supplementary Notes:

Supplementary Note 1. Model ranking and selection criteria

We fitted each model (M_j) described in **Methods**, section ***Mathematical models of B cell dynamics during an immune response*** simultaneously to the time-courses of cell counts of CAR$^+$ MZB cells, CAR$^+$ GCB cells and total B cells from control/CAR and N2KO//CAR mice. The model parameters (expressed as a vector θ) and the errors associated with the empirical measurements in each dataset (σ_i) are then estimated using the Bayesian statistical inference approach. We assumed that the residuals are normally distributed. The likelihood and the probability density for each dataset are then defined as,

$$\begin{aligned} y_i &\sim \text{Normal}(\mu_i, \sigma_i) && \text{[Likelihood]} \\ \mu_i &= M_j(\text{time}_i, \theta) && \text{[Model prediction]} \\ P(y_i|\theta) &= \frac{1}{2\,\pi\,\sigma^2} \exp\left(-\frac{(y_i - \mu_i)^2}{2\,\sigma^2} \right) && \text{[Probability density]} \end{aligned}$$

Supplementary Equation (1)

We defined the 'prior' distributions of model parameters representing our assumptions regarding their values. The Bayesian procedure then updates the priors using the likelihood, to generate posterior distribution ($\hat{\theta}$) of model parameters conditional on the evidence from the data. The input for this procedure is the joint probability densities of three datasets.

$$P(\hat{\theta}|y_1, y_2, y_3) = \frac{P(y_1|\hat{\theta}) \cdot P(y_2|\hat{\theta}) \cdot P(y_3|\hat{\theta}) \cdot P(\theta)}{P(y_1) \cdot P(y_2) \cdot P(y_3)}$$

Supplementary Equation (2)

Models are fitted using the no-U-turn-sampler (*NUTS*) sampler in the Stan programming language, where Parameters are sampled from the joint density $P(\theta)$ following Hamiltonian Monte Carlo algorithm (5). The descriptions of model systems, the prior distributions of parameters and the likelihood definitions are encoded in the Stan language and are published in an open access repository (6).

We compared the support for models using the leave-one-out (LOO) cross validation method, which estimates the expected log point-wise predictive density (*elpd*) for each model M_j – the measure of its out-of-sample prediction accuracy. We estimate the probability density of y_i given the model M_j fitted on the data with observation i excluded → $P(y_i|y_{-i},\, M_j)$. This leave-one-out process is repeated for all $'n'$ observations in the data. The *elpd* estimate and its standard error are then calculated as,

$$\widehat{\mathrm{elpd}^j_{\mathrm{loo}}} = \sum_{i=1}^{n} \mathrm{elpd}^j_{\mathrm{loo,\,i}} = \sum_{i=1}^{n} \log(P(y_i|y_{-i}, M_j)),$$

$$\mathrm{se}(\widehat{\mathrm{elpd}^k_{\mathrm{loo}}}) = \sqrt{\sum_{i=1}^{n} \left(\mathrm{elpd}^k_{\mathrm{loo,\,i}} - \mathrm{elpd}^k_{\mathrm{loo}}/n\right)^2}.$$

Supplementary Equation (3)

We used the *loo-2.0* package in the *Rstan* library to estimated *elpd*, which employs Pareto smoothed importance sampling (PSIS) (7) to approximate LOO cross validation. The estimates of elpd and its standard error were used to rank models using the Pseudo-Bayesian model averaging (BMA) method (8) implemented in the *loo-2.0* package. The model weight implies relative support for each model, analogous to the model weights calculated using Akaike's Information Criterion (AIC) (9-11), and is given as,

$$W_k = \frac{\exp\left(\widehat{\mathrm{elpd}^k_{\mathrm{loo}}} - \frac{1}{2}\mathrm{se}(\widehat{\mathrm{elpd}^k_{\mathrm{loo}}})\right)}{\sum_{k=1}^{K} \exp\left(\widehat{\mathrm{elpd}^k_{\mathrm{loo}}} - \frac{1}{2}\mathrm{se}(\widehat{\mathrm{elpd}^k_{\mathrm{loo}}})\right)}.$$

Supplementary Equation (4)

As the models' weight calculations are based on the *elpd* estimates from LOO cross-validation, it also denotes the confidence in model's ability to predict new data relative to all the other models considered in the analysis. Model weights for all the fitted models are shown in Table 1a of the Results in the main text.

Supplementary Note 2. Defining phenomenological functions to capture the dynamics of precursor populations for the GCB and CAR-expressing MZB cells

Dynamics of Folicular B (FoB) cells: In the branched model, we assumed that total FoB cells differentiate into Marginal Zone B (MZB) cells. We used the following empirical descriptor function to capture the time course of counts of total FoB cells.

$$\phi(t) = \phi_0 \; (1 + e^{-\nu\,(t-b_0)^2})$$ Supplementary equation (5)

We estimated the parameters ϕ_0, ν and b_0 by fitting the supplementary equation (1) to the log-transformed numbers of FoB cells. The fit is shown in the Supplementary Fig. 8.

Dynamics of CAR$^-$ MZB cells: In all our models we considered that activation of CAR$^-$ MZB cells results in CAR expression. The time course of total numbers of CAR$^-$ MZB cells was also modeled using the supplementary equation (5) and the corresponding model fit is shown in the Supplementary Fig. 12.

Supplementary Note 3. Predicting B cell diversification patterns in response to immunization in an adoptive transfer strategy

Our modelling analysis suggests that the branched pathway is a dominant mode of MZB cell generation upon immunization. It is possible that the true model may lie at the intersection of both branched and linear pathways. To untangle their contributions to MZB cell generation during an ongoing immune response, we propose an adoptive transfer experiment. Here we show the simulations of the proposed experiment generated using the parameters derived from r fitting branched and linear models to the TD-immunization data of control/CAR mice.

In this strategy, CAR+ FoB and GCB cells are sorted on day 7 post immunization from congenic control/CAR mice of CD45.1 and CD45.2 backgrounds, respectively. Sorted cells are then co- transferred in equal proportions in wild-type recipient mice (CD45.1 CD45.2 double positive back- ground) that were immunized synchronously with donor mice (Supplementary Fig. 13A). In this system, CAR-expression identifies activated donor-derived B cells. Within the CAR+ MZB cell subset, CD45.1 and CD45.2 expression segregates cells developed from CD45.1+ FoB (either directly or via GCB stage) and CD45.2+ GCB cells, respectively. We generated predictions for the dynamics of CAR+ MZB and GCB cells, considering (i) the branched model, (ii) the linear model, and (iii) a hybrid model in which both branched and linear pathways govern B cell dynamics during immune responses. We assumed 10% grafting efficiency for 106 FoB (CD45.1) and 106 GCB (CD45.2) transferred cells.

We used the median values of parameters estimated from fitting the branched and linear models separately to the CAR expression dynamics in control/CAR mice during the TD-immune response. These parameters are described in detail in the published repository (6). Further, we explored four values of the rate of loss (either by death or differentiation into plasma or memory fates) of CAR+ FoB cells *viz.* ϕ = 0.01, 0.5, 1 and 5 day−1.

Results of the simulations:

(a) **Branched model:** In this model, all CAR^+ MZB cells are generated from FoB $CD45.1^+$ donor cells. $CD45.2^+$ GCB cells do not differentiate into MZ B cells. If the branched model is the only pathway MZB cell generation during immune activation then the fraction of $CD45.1^+$ cells in CAR^+ MZB cell compartment would always equal 100% (Supplementary Fig. 13B).

(b) **Linear model:** In this scenario, CAR^+ MZB cell subset contains both $CD45.1^+$ and $CD45.2^+$ cells. $CD45.2^+$ MZB cells are generated from linear differentiation of transferred $CD45.2^+$ GCB cells. $CD45.1^+$ MZB cells are generated from $CD45.1^+$ GCB cells, which come from differentiation of transferred $CD45.1^+$ FoB cells. Our simulation of the linear model predicts that immediately post transfer donor MZB compartment predominantly contains $CD45.2^+$ cells (Supplementary Fig. 13C), and their fraction declines over time as $CD45.1^+$ MZB cells gradually emerge from $CD45.1^+$ GCB cells. Additionally, the CD45.2 fraction in donor MZB subset approaches 1 with increasing values of the loss rate of CAR^+ FoB cells (Supplementary Fig. 13C).

(c) **Hybrid (branched + linear) model:** In this model, $CD45.1^+$ cells in CAR^+ MZB compartment are generated from $CD45.1^+$ FoB and $CD45.1^+$ GCB cells. $CD45.2^+$ cells in CAR^+ MZB compartment come from linear differentiation of $CD45.2^+$ GCB cells. Our simulation suggests that the dynamics of $CD45.1^+$ and $CD45.2^+$ cells in CAR^+ MZB compartment relies strongly on the loss rate of CAR^+ FoB cells – ϕ. At values of $\phi < 0.7$, the fraction of $CD45.1^+$ cells in the donor MZB compartment are higher than the fraction of $CD45.2^+$ cells. This pattern reverses for values of $\phi \geq 0.7$, and the number of $CD45.2^+$ cells surpass the number of $CD45.1^+$ cells in the CAR^+ MZB compartment (Supplementary Fig. 13D).

These simulations predict distinct quantitative and qualitative outcomes for the branched, linear and hybrid models. Our analysis here suggests that mathematically modeling the data-derived from the proposed co-transfer experiment can effectively untangle the contributions of branched and linear pathways to MZB cell generation during immune responses.

References:

1. Besseyrias V, Fiorini E, Strobl LJ, Zimber-Strobl U, Dumortier A, Koch U, et al. Hierarchy of Notch-Delta interactions promoting T cell lineage commitment and maturation. J Exp Med. 2007;204(2):331-43.

2. Hampel F, Ehrenberg S, Hojer C, Draeseke A, Marschall-Schroter G, Kuhn R, et al. CD19-independent instruction of murine marginal zone B-cell development by constitutive Notch2 signaling. Blood. 2011;118(24):6321-31.

3. Casola S, Cattoretti G, Uyttersprot N, Koralov SB, Seagal J, Hao Z, et al. Tracking germinal center B cells expressing germ-line immunoglobulin gamma1 transcripts by conditional gene targeting. Proc Natl Acad Sci U S A. 2006;103(19):7396-401.

4. Heger K, Kober M, Riess D, Drees C, de Vries I, Bertossi A, et al. A novel Cre recombinase reporter mouse strain facilitates selective and efficient infection of primary immune cells with adenoviral vectors. Eur J Immunol. 2015;45(6):1614-20.

5. Team SD. Stan modeling language user's guide and reference manual, version 2.29 Stan. 2022 [Available from: https://mc-stan.org.

6. Rane S. Notch2 controls developmental fate choices between germinal center and marginal zone B cells upon immunization [Internet]. 2024. Available from: https://zenodo.org/records/10475721.

7. Vehtari A, Gelman A, Gabry J. Practical Bayesian model evaluation using leave-one-out cross-validation and WAIC. Statistics and Computing. 2016;27:1413–32.

8. Yao Y, Vehtari A, Simpson D, Gelman A. Using Stacking to Average Bayesian Predictive Distributions (with Discussion). Bayesian Anal. 2018;13(3): 917-1007.

9. Akaike H. On the Likelihood of a Time Series Model. Journal of the Royal Statistical Society Series D (The Statistician). 1978;27(3/4):217-35.

10. Burnham KP, Anderson DR. Model Selection and Inference - A Practical Information-Theoretic Approach: Springer New York, NY; 2013. 355 p.

11. Wagenmakers E-J, Farrell S. AIC model selection using Akaike weights. Psychonomic Bulletin & Review. 2004;11(1):192–6.
