# Supplementary material for: Notch2 controls developmental fate choices between germinal center and marginal zone B cells upon immunization
Source: Nat Commun. 2024 Mar 4;15:1960. doi: 10.1038/s41467-024-46024-1 (PMC10912316; doi:10.1038/s41467-024-46024-1)
Supplement: Supplementary file 3 — Reporting Summary [file 41467_2024_46024_MOESM3_ESM.pdf]

nature portfolio

Corresponding author(s): Dr. Ursula Zimber-Strobl

Last updated by author(s): Jan 18, 2024

# Reporting Summary

Nature Portfolio wishes to improve the reproducibility of the work that we publish. This form provides structure for consistency and transparency in reporting. For further information on Nature Portfolio policies, see our Editorial Policies and the Editorial Policy Checklist.

## Statistics

For all statistical analyses, confirm that the following items are present in the figure legend, table legend, main text, or Methods section.

| n/a | Confirmed | |
|---|---|---|
| ☐ | ☒ | The exact sample size (*n*) for each experimental group/condition, given as a discrete number and unit of measurement |
| ☐ | ☒ | A statement on whether measurements were taken from distinct samples or whether the same sample was measured repeatedly |
| ☐ | ☒ | The statistical test(s) used AND whether they are one- or two-sided<br>*Only common tests should be described solely by name; describe more complex techniques in the Methods section.* |
| ☒ | ☐ | A description of all covariates tested |
| ☒ | ☐ | A description of any assumptions or corrections, such as tests of normality and adjustment for multiple comparisons |
| ☐ | ☒ | A full description of the statistical parameters including central tendency (e.g. means) or other basic estimates (e.g. regression coefficient) AND variation (e.g. standard deviation) or associated estimates of uncertainty (e.g. confidence intervals) |
| ☐ | ☒ | For null hypothesis testing, the test statistic (e.g. *F*, *t*, *r*) with confidence intervals, effect sizes, degrees of freedom and *P* value noted<br>*Give P values as exact values whenever suitable.* |
| ☐ | ☒ | For Bayesian analysis, information on the choice of priors and Markov chain Monte Carlo settings |
| ☒ | ☐ | For hierarchical and complex designs, identification of the appropriate level for tests and full reporting of outcomes |
| ☒ | ☐ | Estimates of effect sizes (e.g. Cohen's *d*, Pearson's *r*), indicating how they were calculated |

*Our web collection on statistics for biologists contains articles on many of the points above.*

## Software and code

Policy information about availability of computer code

| | |
|---|---|
| Data collection | Diva Software BD FACS DIVA V8.0.1<br>Leica Application Suite X (LAS X) Software, Version 4<br>Immunospot Version 5.0<br>Infinite F200 PRO, i-control software, Version 3.37 |
| Data analysis | R Studio Version 4.1.1<br>CmdStan Version 2.31<br>GraphPad PRism Version 9.5.1<br>Immunospot Version 5.0<br>MS Excel 2016<br>ImageJ 1.50e<br>FlowJo Version 10.7.2 |

For manuscripts utilizing custom algorithms or software that are central to the research but not yet described in published literature, software must be made available to editors and reviewers. We strongly encourage code deposition in a community repository (e.g. GitHub). See the Nature Portfolio guidelines for submitting code & software for further information.

## Data

Policy information about availability of data

All manuscripts must include a data availability statement. This statement should provide the following information, where applicable:

- Accession codes, unique identifiers, or web links for publicly available datasets
- A description of any restrictions on data availability
- For clinical datasets or third party data, please ensure that the statement adheres to our policy

Already existing RNA expression data from our group analyzed in this study are available at ENA under the accession code PRJEB35207 (Suppl.Figure 1C) or at Zenodo under the DOI 10.5281/zenodo.10491127 (Figure 1C). Graphs from Suppl. Figure 8B were produced with data derived from the ImmGen.org databrowser "Gene Skyline – RNAseq" (http://rstats.immgen.org/Skyline/skyline.html). All other source data are provided with this paper as Excel file. Code defining all our models and scripts defining our plotting and statistical routines are available at a Github repository integrated in the Zenodo records and published with the DOI: 10.5281/zenodo.10472653.

## Research involving human participants, their data, or biological material

Policy information about studies with human participants or human data. See also policy information about sex, gender (identity/presentation), and sexual orientation and race, ethnicity and racism.

| | |
|---|---|
| Reporting on sex and gender | N/A |
| Reporting on race, ethnicity, or other socially relevant groupings | N/A |
| Population characteristics | N/A |
| Recruitment | N/A |
| Ethics oversight | N/A |

Note that full information on the approval of the study protocol must also be provided in the manuscript.

# Field-specific reporting

Please select the one below that is the best fit for your research. If you are not sure, read the appropriate sections before making your selection.

☒ Life sciences ☐ Behavioural & social sciences ☐ Ecological, evolutionary & environmental sciences

For a reference copy of the document with all sections, see nature.com/documents/nr-reporting-summary-flat.pdf

# Life sciences study design

All studies must disclose on these points even when the disclosure is negative.

**Sample size**

Sample sizes were determined based on previous experiments and preliminary data. No statistical method was used to predetermine sample size. Our goal was to analyze at least 6 animals per group. In cases, in which differences were pronounced enough to achieve significance with smaller sample sizes, adjustments were made accordingly. In general, we analyzed a minimum of 4 samples per genotype and treatment.

There were some exceptions. In Fig. 4a (5h time point), the sample size was n=2 and was excluded from the statistical analysis. In Fig. 7c, Supplementary Fig. 2c, Supplementary Fig. 4, ELISpots in supplementary Fig. 7a n=3 mice were analysed. In most cases, the data presented in these figures are from experiments performed during the first revision phase. During this time, we only had a limited number of mice available and were therefore unable to perform all the experiments requested by the reviewers with higher numbers of mice. Despite the smaller number of mice, our analyses still yielded significant results or, at least, clear trends, as the biological differences between the samples were sufficiently robust.

For mathematical modeling, values were collected from at least two independent immunization rounds, with the exception of day 17, where one immunization round with 3 mice was performed. At each time point, a minimum of 3 mice per genotype were utilized for data gathering.

**Data exclusions**

No data exclusions were done.

**Replication**

All in vivo experiments were performed in at least two or more independent immunization rounds. In vitro experiments were performed at least in duplicates with the indicated numbers of mice. Replications of in vivo and in vitro experiments were successful.

**Randomization**

For all in vivo and in vitro studies, mice were allocated in cohorts based on genotype and/or time points after NP-CGG antigen injection (immunization) and no randomization was performed. Mice used in the study were maintained at minimally perturbative conditions. Control and analytical mice were always analysed in parallel and predominantly used in an age-matched manner, when possible. The experiments were repeated with different individual animals and several rounds of independent immunizations in several experiments.

Blinding

In all experiments, the investigators were not blinded, as phenotype observations in flow cytometry, ELISA/ELISpot and in microscopy were clearly visible and consistent with the genotype of the analytical animal(s). Flow cytometry data analyses were performed unbiased, with consistent gating of lymphocyte populations for all genotypes and time points.

# Reporting for specific materials, systems and methods

We require information from authors about some types of materials, experimental systems and methods used in many studies. Here, indicate whether each material, system or method listed is relevant to your study. If you are not sure if a list item applies to your research, read the appropriate section before selecting a response.

## Materials & experimental systems

| n/a | Involved in the study |
|---|---|
| ☐ | ☒ Antibodies |
| ☒ | ☐ Eukaryotic cell lines |
| ☒ | ☐ Palaeontology and archaeology |
| ☐ | ☒ Animals and other organisms |
| ☒ | ☐ Clinical data |
| ☒ | ☐ Dual use research of concern |
| ☒ | ☐ Plants |

## Methods

| n/a | Involved in the study |
|---|---|
| ☒ | ☐ ChIP-seq |
| ☐ | ☒ Flow cytometry |
| ☒ | ☐ MRI-based neuroimaging |

## Antibodies

Antibodies used

For FACS:
1. Anti-Human CD2 APC monoclonal antibody (clone RPA-2.10), Invitrogen eBioscience, Cat# 17-0029-42; dilution 1:100, RRID: AB_10805740
2. Anti-Human CD2 PE monoclonal antibody (clone RPA-2.10), Invitrogen eBioscience, Cat# 12-0029-42; dilution 1:100, RRID: AB_10670621
3. Anti-Human CD2 FITC REAfinity™ Antibody (clone REA972), Miltenyi Biotec, Cat# 130-116-251; dilution 1:50, RRID: AB_2727364
4. Anti-human CD2 Pe Vio770 REAfinity™ Antibody (clone REA972), Miltenyi Biotec, Cat# 130-116-254; dilution 1:100, RRID: AB_2727364
5. Anti-Human CAR (E1-1) FITC (clone E1-1), Santa Cruz, Cat# sc-56892 FITC, dilution 1:25, RRID: N/A (product has been discontinued by Santa Cruz end of 2022, no manufacturer data available. For validation, please refer to following publications: https://doi.org/10.3390/cells11050841; https://doi.org/10.1182/blood.2018880138; https://doi.org/10.1038/s41598-018-31713-x)
6. Anti-Mouse CD1d AF647 (clone 1B1), Biolegend, Cat#123511; dilution 1:200, RRID: AB_1236539
7. Anti-Mouse TACI APC (clone ebio8F10-3), eBioscience, Cat#17-5942; dilution 1:100, RRID: AB_842758
8. Anti-Mouse CD86 APC (clone GL1), BD Biosciences, Cat# 561964; dilution 1:100, RRID: AB_2075114
9. Anti-Mouse CD184 (CXCR4) PE monoconal antibody (clone 2B11), eBioscience, Cat# 12-9991-82; dilution 1:400, RRID: AB_891391
10. Anti-Mouse CD38 Pe Vio770 REAfinity™ Antibody (clone REA616), Miltenyi Biotec, Cat# 130-125-522; dilution 1:100 (1:50 for IC), RRID: AB_2802049
11. Anti-Mouse CD21/CD35 BV421 (clone 7G6), BD Biosciences, Cat# 562756; dilution 1:200, RRID: AB_2737772
12. Anti-Mouse CD97 (AA4.1) PE (clone AA4.1), eBioscience, Cat# 12-5892; dilution 1:200, RRID: AB_466018
13. Anti-Mouse CD138 BV421 (clone 281-2), BD Biosciences, Cat# 562610; dilution 1:200, RRID: AB_11153126
14. Anti-Mouse CD95 BV421 (clone Jo2), BD Biosciences, Cat# 562633; dilution 1:200 (1:100 for IC), RRID: AB_2737690
15. Anti-Mouse CD95 PE (clone Jo2), BD Biosciences, Cat# 554258; dilution 1:300, RRID: AB_395330
16. Anti-Mouse Bcl-6 Alexa Fluor® 647 (clone K112-91), BD Biosciences, Cat# 561525; dilution 1:50, RRID: AB_10898007
17. Anti-Mouse PRDM1/Blimp-1 PE (clone 6D3), Santa Cruz, Cat# sc-47732 PE; dilution 1:50, For validation statements and relevant citations provided by manufacturer, please see https://datasheets.scbt.com/sc-47732.pdf.
18. Anti-Mouse IRF4 eFluor 660 (clone 3E4), eBioscience, Cat# 50-9858-82; dilution 1:100, RRID: AB_2574393
19. Anti-Mouse IRF4 PE (clone 3E4), eBioscience, Cat# 12-9858-82; dilution 1:500, RRID: AB_10852721
20. Anti-Mouse Notch2 PE (clone HMN2-35), Biolegend, Cat# 130707; dilution 1:80, RRID: AB_1227725
21. Anti-Mouse CD43 Biotin (clone S7), BD Biosciences, Cat# 553269; dilution 1:350, RRID: AB_2255226
22. Anti-Mouse CD43 Bv421 (clone S7), BD Biosciences, Cat# 562958; dilution 1:200, RRID: AB_2665409
23. Anti-Mouse CD19 PE Vio770 (clone REA749), Miltenyi Biotec, Cat# 130-112-037; dilution 1:200, RRID: AB_2655830
24. Anti-Mouse CD19 BV510 (clone 1D3), BD Biosciences, Cat# 562956; dilution 1:200, RRID: AB_2737915
25. Anti-Mouse CD45R/B220 PerCP (clone RA3-6B2), BD Biosciences, Cat# 553093; dilution 1:100, RRID: AB_394622
26. Anti-Mouse CD45R/B220 APC (clone RA3-6B2), BD Biosciences, Cat# 553092; dilution 1:250, RRID: AB_398531
27. Anti-Mouse CD45R/B220 PE (clone RA3-6B2), BD Biosciences, Cat# 553090; dilution 1:350, RRID: AB_394619
28. Anti-Mouse CD45R/B220 FITC (clone RA3-6B2), BD Biosciences, Cat# 553088; dilution 1:200, RRID: AB_394618
29. Streptavidin PerCP (clone N/A), BD Biosciences, Cat# 554064; dilution 1:100, RRID: AB_2336918
30. Streptavidin APC (clone N/A), BD Biosciences, Cat# 554067; dilution 1:400, RRID: AB_10050396
31. Anti-Mouse CD23 PE (clone B3B4), BD Biosciences, Cat# 553139; dilution 1:200, RRID: AB_394654
32. Anti-Mouse CD23 Alexa Fluor® 647 (clone B3B4), BD Biosciences, Cat# 562826; dilution 1:100, RRID: AB_2737821
33. Anti-Mouse CD23 PE-Cy7 (clone B3B4), BD Biosciences, Cat# 562825; dilution 1:200, RRID: AB_2737820
34. Anti-Mouse IgM APC (clone II/41), BD Biosciences, Cat# 562032; dilution 1:100, RRID: AB_398464
35. Anti-Mouse IgM PE-Cy7 (clone R6-60.2), BD Biosciences, Cat# 552867; dilution 1:300, RRID: AB_394500
36. Anti-Mouse IgD Biotin (clone 11-26c), eBioscience, Cat# 13-5993-82; dilution 1:350, RRID: AB_466860
37. Anti-mouse IgG1 PE (clone A85-1), BD Biosciences, Cat# 550083; dilution 1:800, RRID: AB_393553
38. Anti-mouse Ki-67 PE monoclonal antibody (clone SolA15), eBioscience/Invitrogen, Cat# 12-5698-82; dilution 1:500, RRID:

AB_11150954
39. Anti-mouse CD54 (ICAM-1) Biotin (clone 3E2), BD Biosciences, Cat# 553251; dilution 1:200, RRID: AB_394733
40. Anti-mouse CD275 (ICOS-L) PE monoclonal antibody (clone HK5.3), eBioscience/Invitrogen, Cat# 12-5985-82; dilution 1:100, RRID: AB_466094
41. Anti-mouse CD80 PE monoclonal antibody (clone 16-10A1), eBioscience/Invitrogen, Cat# 12-0801-82; dilution 1:400, RRID: AB_465752
42. Anti-mouse CD86 PeCy5 monoclonal antibody (clone GL1), eBioscience/Invitrogen, Cat# 15-0862-82; dilution 1:200, RRID: AB_468778
43. NP (4-Hydroxy-3-nitrophenylacetyl hapten) PE, conjugation ratio 19 (NP19-PE), LGC Biosearch Technologies, Cat# N-5070-1-BS; dilution 1:100, For validation statements and relevant citations provided by manufacturer, please see https://shop.biosearchtech.com/immunochemicals/hapten-conjugates/np-pe-%28phycoerythrin%29/p/N-5070-1#specifications
44. Anti-Mouse CD184 (CXCR4) BDHorizon BV510 (clone 2B11/CXCR4), BD Biosciences, Cat# 563468; dilution 1:200, RRID: AB_2738225
45. Rabbit anti-Hes1 unconjugated monoclonal antibody (clone D6P2U), Cell Signaling, Cat# 11988S; dilution 1:100, For validation statements and relevant citations provided by manufacturer, please see https://www.cellsignal.com/datasheet.jsp?productId=11988&images=1&size=A4
46. Goat anti-Rabbit IgG (H+L) PE cross-adsorbed secondary antibody, Invitrogen, Cat# P-2771MP; dilution 1:100, RRID: AB_2539845
47. Annexin V Apoptosis Detection Kit I PE, BD Biosciences, Cat# 559763; dilution 5µl per 1 million cells in 100µl, RRID: AB_2869265
48. CellTrace™ Far Red Cell Proliferation Kit (APC), for flow cytometry, Invitrogen, Cat# C34564; dilution 1:1000, For validation statements, images and relevant citations provided by manufacturer, please see https://www.thermofisher.com/order/catalog/product/de/en/C34564

For ELISA/ELISpot:
1. Anti-Mouse IgM Biotin (clone R6-60.2), BD Biosciences, Cat# 553406; dilution 1:500, RRID: AB_394843 (for ELISpot)
2. Anti-Mouse IgG1 Biotin (clone A85-1), BD Biosciences, Cat# 553441; dilution 1:500, RRID: AB_394861 (for ELISA and ELISpot)
3. Anti-Mouse IgG3 Biotin (clone R40-82), BD Bioscience, Cat# 553401; dilution 1:500, RRID: AB_394838 (For ELISpot)
4. Anti-Mouse IgM HRP (clone 1B4B1), Southern Biotech, Cat# 1140-05; dilution 1:5000, RRID: AB_2794629 (for ELISA)
5. Horseradish Peroxidase Avidin D (Av-HRP) (clone N/A), Vector, Cat# A-2014-5; dilution 1:2000, (for ELISA and ELISpot); For specifications, documents and technical information, please refer to https://vectorlabs.com/products/avidin-d-horseradish-peroxidase-concentrate-elisa;

For Histology (IF/IHC):
1. Anti-Laminin (clone L9393), Sigma-Aldrich, Cat# L9393; dilution 1:100, RRID:AB_477163
2. Anti-Human CD2 Biotin (clone RPA-2.10), BD Biosciences, Cat# 555325; dilution 1:50, RRID:AB_395732
3. Anti-Mouse CD90.2 (Thy1.2) Biotin (clone 30-H12), BD Biosciences, Cat# 553011; dilution 1:100, RRID:AB_394549
4. Anti-Mouse CD45R/B220 APC (clone RA3-6B2), BD Biosciences, Cat# 553092; dilution 1:500, RRID:AB_398531
5. Anti-Mouse T- and B-Cell Activation Antigen (GL7) FITC (clone GL7), BD Biosciences, Cat# 553666; dilution 1:100, RRID:AB_394981
6. Anti-Mouse T- and B-Cell Activation Antigen (GL7) Alexa Fluor® 647 (clone GL7), BD Biosciences, Cat# 561529; dilution 1:100, RRID:AB_10716056
7. Anti-Metallophilic Macrophages (MOMA-1) Biotin (clone MOMA-1), Abcam, Cat# ab51814; dilution 1:100, For validation statement and relevant citations, please refer to https://www.abcam.com/products/primary-antibodies/biotin-metallophilic-macrophages-antibody-moma-1-ab51814.html and the archived datasheet_51814 on that web page;
8. Anti-Mouse Irf4 purified antibody (clone 3E4), eBioscience, Cat# 14-9858-80; dilution 1:100, For validation statement and relevant citations, please refer to https://www.thermofisher.com/antibody/product/IRF4-Antibody-clone-3E4-Monoclonal/14-9858-82;
9. Anti-Mouse IgM (clone 1020-01), Southern Biotech, Cat# 1020-01; dilution 1:100, RRID:AB_2794197
10. Anti-Goat IgG Alexa Fluor 647, Invitrogen, Cat# A-21469; dilution 1:500, RRID:AB_2535872
11. Anti-Rat IgG Alexa Fluor 488, Jackson ImmunoResearch, Cat# 112-545-003; dilution 1:500, RRID: AB_2338351
12. Anti-rabbit IgG Cyanine Cy3, Jackson ImmunoResearch, Cat# 111-165-003; dilution 1:500, RRID: AB_2338000
13. Anti-Rat IgG Alexa Fluor 647, Jackson ImmunoResearch, Cat# 112-605-167; dilution 1:500, RRID: AB_2338404
14. Anti-Rabbit IgG Peroxidase, Sigma-Aldrich, Cat# A0545; dilution 1:200, RRID:AB_1645286
15. Streptavidin, Alexa Fluor™ 594, Invitrogen, Cat# S11227; dilution 1:500, For validation statement, datasheets and relevant citations, please refer to https://www.thermofisher.com/order/catalog/product/de/en/S11227

Validation

All antibodies show reactivity to mouse (or for CD2 to human), as needed for the study. All purchased antibodies were chosen under careful consideration of the manufacturer's validation data and QC reports about antigen specificity, application and species reactivity. This is visible directly on the manufacturers' web pages to each product. All validation data, relevant citations, QC reports, datasheets and images of the antibodys' profile on tested cells can be found by a internet web-search of the RRID AB_XXXXXX (6-digit) number provided above for each antibody. For several antibodies (for histology and flow cytometry) an RRID identification number was not available by the manufacturer, therefore, https links were given which lead directly to these information.

The working dilutions of all antibodies, as used in the experiments of this study, are listed above and in the Supplementary Table 1 found in the Supplementary Information PDF File.

## Animals and other research organisms

Policy information about studies involving animals; ARRIVE guidelines recommended for reporting animal research, and Sex and Gender in Research

Laboratory animals

Mouse: Notch2ICSTOPfl (Hampel et al., 2011)
Mouse: R26/CAG-CARd1STOPfl (Heger et al., 2015)
Mouse: Notch2fl/fl (Besseyrias et al., 2007)
Mouse: Cy1-cre (Casola et al., 2006)
Mouse: BALB/c, Charles River Germany, JAX Stock No 000651
Mouse: CBF:H2B-Venus (Notch-reporter) (Nowotschin et al., 2013), purchased from Jackson Laboratories, JAX Stock No 020942

Male and female mice at the age of 10-18 weeks were used for all experiments. All mice were on a pure Balb/c background, except for CBF:H2B-Venus reporter mice, which were backcrossed after purchase for 5 generations on a Balb/c background. All animal experiments were performed in compliance with the German Animal Welfare Law and were approved by the Institutional Committee on Animal Experimentation and the Government of Upper Bavaria.
The animals were kept in the laboratory animal rooms under specified pathogen-free (SPF) conditions with a 12/12-hour light cycle. The animal rooms are fully air-conditioned, the set-points are set to 20 - 24°C temperature and 45 - 65 % humidity according to Annex A of the European Convention 2007/526 EC. The rooms are equipped with individually ventilated cage systems (e.g. Tecniplast Greenline GM 500, 501 cm² floor area, or BioZone-IVC type II, 370 cm² floor area). The maximum stocking densities comply with Annex III of Directive 2010/63/EU. The cages are equipped with laboratory animal bedding (wood fiber/wood chips, e.g. Lignocel Select Fine, SAFE). The animals were given sterile filtered water and a standard rodent diet (e.g. Altromin 1314) ad libitum. Control and experimental mice were bred separately, but were maintained in the same area of the mouse facility. The mice were euthanized either with CO2 or by cervical dislocation. The QuietekTM-apparatus was used for euthanasia with CO2 to ensure that the CO2 was supplied with the correct flow rate up to the correct filling volume.

Wild animals: N/A

Reporting on sex: Findings from this study do not apply to only one sex. Sex of mice was not considered as a decisive factor in the study design and methods. Both male and female mice were used for the experiments, data were analyzed without consideration of the animals' sex. To ensure a more balanced distribution of both male and female animals throughout the study, control and analytical mice were sex-matched in experiments, whenever possible.
A detailed overview of the sex and age of each mouse is provided in the source data file (Excel) for each Figure showing experimental data with mice.

Field-collected samples: N/A

Ethics oversight: The experiments were performed in compliance with the German Animal Welfare Law and were approved by the Institutional Committee on Animal Experimentation and the government of Upper Bavaria.

Note that full information on the approval of the study protocol must also be provided in the manuscript.

## Plants

Seed stocks: N/A

Novel plant genotypes: N/A

Authentication: N/A

## Flow Cytometry

### Plots

Confirm that:

☒ The axis labels state the marker and fluorochrome used (e.g. CD4-FITC).

☒ The axis scales are clearly visible. Include numbers along axes only for bottom left plot of group (a 'group' is an analysis of identical markers).

☒ All plots are contour plots with outliers or pseudocolor plots.

☒ A numerical value for number of cells or percentage (with statistics) is provided.

### Methodology

Sample preparation: Single cell suspensions were prepared from the spleen and bone marrow (BM) of mice. Following organ extraction, the SP were preserved in 1% B cell medium on ice (BCM; 1x RPMI 1640 supplemented with 1% (v/v) heat-inactivated fetal calf serum (FCS) (PAA Cell culture Company), 100 U/ml penicillin, 100 µg/ml streptomycin, 1 mM sodium pyruvate, 2 mM L-glutamine, 1x non-essential amino acids, and 50 µM β-mercaptoethanol (all purchased from Gibco)). BM was collected by flushing the femur and tibia bones with 1% BCM and a cannula. For the preparation of single cell suspensions, SP was mashed through a 70 µM strainer. Following centrifugation at 1200 rpm for 10 min at 4 degrees, spleen and BM cell suspensions were lysed for 3 min at room temperature in 1 mL of red blood lysis buffer (RBC) buffer (1x, eBioscience) to remove erythrocytes. The reaction was terminated by addition of 12 mL 1% BCM and centrifugation under the same conditions. Finally, cells from the spleen and BM were resuspended in 1 mL 1% BCM and were counted to establish the cell number for Flow Cytometry experiments.
Surface staining of lymphocytes with the corresponding antibodies in MACS buffer (Miltenyi) was performed on ice for 25 min. To omit dead cells from the analysis, cells were stained for 20 min on ice with LIVE/DEAD Fixable Blue Dead Cell Stain Kit (Invitrogen) prior to extracellular antibody staining. For intracellular staining, cells were fixed with 2% paraformaldehyde (1:2

| | |
|---|---|
| | PBS-diluted Histofix, Carl Roth) for 10 min at room temperature and permeabilized in ice-cold 100% methanol for 10 min on ice. Cells were incubated for 1 h at room temperature in the dark with the corresponding antibodies. Dead cells were excluded by staining for 5 min on ice with LIVE/DEAD Fixable Blue Dead Cell Stain Kit (Invitrogen) before fixation. |
| Instrument | LSRII FACS Fortessa (BD Biosciences) |
| Software | FlowJo Version 9 and Version 10.7.2 |
| Cell population abundance | For the in-vitro experiment measuring Notch2 surface expression on purified FoB cells, the FoB cells were isolated with the MZB and FoB Cell Isolation Kit (Miltenyi Biotech). The enrichment of mature B cells was checked in a preliminary staining prior to the experiment by a CD19 vs. Thy1.2 co-staining (purified B cells were identified as CD19+ Thy1.2-, data not shown here). The purity of FoB cells (no MZB cell contamination) was reassured in a subsequent staining for CD23 and CD21 within the CD19+ Thy1.2- cell population (preliminary staining, data not shown). FoB cells were herein identified as CD23+ CD21low/neg. The purity of the used FoB cells was always at least 96%.<br>For the plasmablast differentiation in vitro assay, total naïve B cells were isolated from splenic cell suspensions with the CD43 depletion B cell Isolation Kit (Miltenyi). The enrichment and purity of B cells was ensured in a CD19 vs. CD43 surface staining (B cells were identified as CD19+CD43-, pre-experiment staining for purity, data not shown). The purity of B cells reached >95% for all experiments. |
| Gating strategy | Representative gating strategies (refering to most flow cytometry data in this manuscript) for surface and intracellular cell stainings to distinguish single living B lymphocytes and their subpopulations, reporter-expressing and reporter-negative cells, as well as plasmablasts and plasma cells live lymphocytes, have been provided in Supplementary Figure 14a-e.<br>Otherwise important gating strategies to identify specific B cell subpopulations (e.g. GC B cells, IgG1-switched cells, light and dark zone GC B cells etc.) are always shown in the contour or pseudocolor plots in each figure containing flow cytometry analyses. In addition, detailed information about the proper identification of cells using their differential extracellular or intracellular marker expression are described in all figure legends.<br>For the mathematical modelling part of the manuscript, specific sequential gating of cell subsets was used. The full hierarchical gating strategy for this part is therefore shown in a separate Supplementary Figure 11. |

☒ Tick this box to confirm that a figure exemplifying the gating strategy is provided in the Supplementary Information.
